# Nonlinear ion drift-diffusion memristance description of TiO_2_ RRAM devices†

**DOI:** 10.1039/d0na00195c

**Published:** 2020-04-21

**Authors:** Sahar Alialy, Koorosh Esteki, Mauro S. Ferreira, John J. Boland, Claudia Gomes da Rocha

**Affiliations:** School of Chemistry, Trinity College Dublin Dublin 2 Dublin Ireland; Centre for Research on Adaptive Nanostructures and Nanodevices (CRANN), Advanced Materials and Bioengineering Research (AMBER) Research Centre, Trinity College Dublin Dublin 2 Dublin Ireland; Department of Physics and Astronomy, University of Calgary 2500 University Drive NW Calgary Alberta T2N 1N4 Canada claudia.gomesdarocha@ucalgary.ca; School of Physics, Trinity College Dublin Dublin 2 Dublin Ireland

## Abstract

The nature and direction of the hysteresis in memristive devices is critical to device operation and performance and the ability to realise their potential in neuromorphic applications. TiO_2_ is a prototypical memristive device material and is known to show hysteresis loops with both clockwise switching and counter-clockwise switching and in many instances evidence of negative differential resistance (NDR) behaviour. Here we study the electrical response of a device composed of a single nanowire channel Au–Ti/TiO_2_/Ti–Au both in air and under vacuum and simulate the *I*–*V* characteristics in each case using the Schottky barrier and an ohmic-like transport memristive model which capture nonlinear diffusion and migration of ions within the wire. The dynamics of this complex charge conduction phenomenon is obtained by fitting the nonlinear ion-drift equations with the experimental data. Our experimental results support a nonlinear drift of oxygen vacancies acting as shallow donors under vacuum conditions. Simulations show that dopant diffusion under bias creates a depletion region along the channel which results in NDR behaviour, but it is overcome at higher applied bias due to oxygen vacancy generation at the anode. The model allows the motion of the charged dopants to be visualised during device operation in air and under vacuum and predicts the elimination of the NDR under low bias operation, in agreement with experiments.

## Abstract 2

The nature and direction of the hysteresis in memristive devices is critical to device operation and performance and the ability to realise their potential in neuromorphic applications.

## Graphical abstract

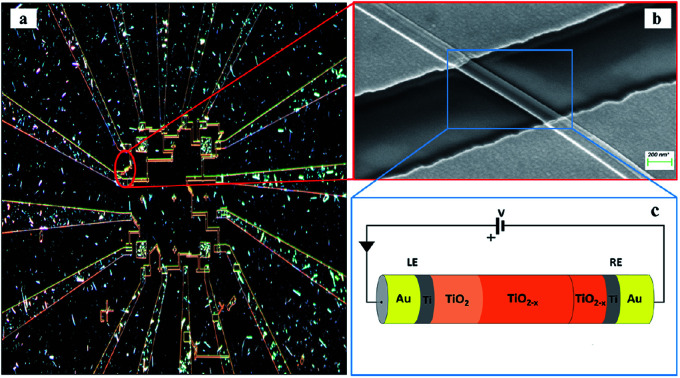

Resistive Random Access Memory (RRAM) devices have recently attracted a great deal of attention due to their superior characteristics that include a simple metal–insulator–metal (MIM) structure, high-density integration, and fast write/erase operation.^1,2^ Resistive switching effects have been observed in many types of material systems. Binary transition metal oxides such as NiO,^3^ Cu_*x*_O (ref. 4) and TiO_*x*_ (ref. 5) are emerging candidates for non-volatile RRAM technologies. However, TiO_2_ remains the prototypical device material and has been the focus of many research studies since it was predicted to enable a specific type of RRAM operation known as memristance.^6–8^ Although many features of memristance behaviour can be described by ion-drift-based memristor models,^6,9–11^ it can be challenging to fit these models to capture the details found in experiments. Numerous current conduction mechanisms^5,12–17^ have been proposed to explain the microscopic memristive features in a wide range of TiO_2_-based devices, but as yet the nature of clockwise (CW) switching and counter-clockwise (CCW) switching of TiO_2_ RRAM is not well understood. Yang *et al.* reported reversible bipolar resistive switching in a Pt/TiO_2_/Pt two-terminal nanodevice, which was modelled as a memristor in parallel with a rectifier.^18,19^ The proposed switching mechanism involved a modification of the Schottky-like barrier at a Pt/TiO_2_ interface caused by the drift of positively charged oxygen vacancies 
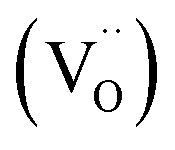
 under an applied electric field.^18^ TiO_2_ is known to have an intrinsic population of oxygen vacancies which behave as shallow n-type dopants that transform the TiO_2_ with a wide band gap (3.3 eV) into an electrically conducting semiconductor. The concentration of 
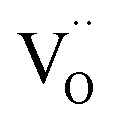
 at the interface can determine the electrical transport and current conduction mechanism behaviour^19^ of the device. The 
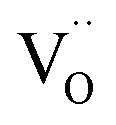
 doping level may also determine whether blocking, tunnelling, and/or rectifying (Schottky-like) contacts are formed. The degree of band bending, barrier height and width depend on the net charges at the interface and the distribution of their energy states in the semiconductor band gap.^13^ Here we describe the surprisingly complex electrical behaviour of a simple TiO_2_ RRAM device under both air and vacuum and apply an extended ion-drift memristance model that accurately describes the device response and provides new insights into its conduction mechanisms.

In this study, the *I*–*V* characteristic of a two-terminal single nanowire Au–Ti/TiO_2_/Ti–Au as a promising RRAM device is investigated. Measurements were performed at distinct temperature ranges with the device placed under vacuum and air conditions. CW switching behaviour is observed under vacuum conditions and the hysteresis loops exhibited a negative differential resistance (NDR) region that can be overcome at higher applied bias due to oxygen vacancy generation at the anode.^20–22^ In comparison to the widely studied Pt/TiO_2_/Pt memristive structures, our Au–Ti/TiO_2_/Ti–Au device simply offers another memristive fingerprint (*I*–*V* hysteresis) option with distinct voltage thresholds which can be used in SET/RESET operations in RRAM devices. In a Pt/TiO_2_/Pt structure, the switching mechanism involves a modification of the Schottky barrier at a Pt/TiO_2_ interface caused by the drift of positively charged oxygen vacancies under an applied electric field. TiO_2_ has an intrinsic population of oxygen vacancies that behave as an n-type dopant and transform the TiO_2_ with a wide band gap (∼3 eV) into an electrically conductive material. Therefore, the concentration of oxygen vacancies at the interface rules the electrical transport and conduction mechanisms of the device. By interfacial engineering of the contacts, we are probing other ways of controlling its conducting or rectifying properties as they affect the characteristics of the Schottky barrier and the distribution of oxygen doping levels at the contacts. The reported experimental Schottky barrier heights for Au/TiO_2_ interfaces lie in the range of 0.9–1.2 eV.^23^ Ti has a work function of typically ∼4.3 eV,^13^ which is higher than the TiO_2_ work function (∼3.2 eV), enabling the formation of a Schottky barrier at the Ti/TiO_2_ interface. In our devices, Ti was used as an adhesion layer and it is regarded as a chemically reactive contact that will reduce the TiO_2_ and create a locally high concentration of oxygen vacancies near the Ti/TiO_2_ interface at sufficiently high voltages. In other words, the Ti layer adds an additional degree of band bending and interface states that alter the properties of the Schottky barrier. The characteristics of the Schottky barrier are structure dependent, and modifying the interface structure at the metal–semiconductor contact will ensure broad implementation of memristive materials in various digital applications. To capture the wealth of resistive switching phenomena observed in our Au–Ti/TiO_2_/Ti–Au *I*–*V* curves, we used exponential ion-drift equations^24–26^ capable of describing ion-migration dynamics in an effective way by means of nonlinear fitting methods^27^ applied to the experimental data. The model shows that two or more different field-driven internal state variables better reproduce the experimental data and that it is necessary to include diffusion effects in addition to simple ion drift induced by an applied electric field.

## 1 Methods

Single nanowire devices Au–Ti/TiO_2_/Ti–Au were fabricated using a three-step process involving UV lithography, TiO_2_ nanowire spray deposition, and electron beam lithography (EBL).^16,17^ Devices were fabricated on silicon substrates with 200 nm oxide thickness. As the first step to define the contact pads, UV lithography was used to produce 150 μm^2^ Ti/Au metal contact pads with a thickness of 5/30 nm, respectively. Dilute solutions of TiO_2_ wires (EMFUTUR) were dispersed in isopropanol (IPA) and deposited on the substrate using a hand-spray. The physical dimensions of the TiO_2_ nanowires were between 50 and 100 nm in diameter and 50 μm in length. The metal contacts on the wires were defined by EBL, and Au metal contacts of 80 nm thickness were drawn to each individual nanowire such that only the Ti metal was in contact with the wires. A Keithley 4200-SCS parameter analyser was used to carry out the electrical measurements (*I*–*V*) on the single TiO_2_ nanowire devices using a 2-point probe setup. Electroforming steps were carried out by applying 5 V with a 100 nA compliance for approximately 10 minutes. Typical triangle wave voltage sweeps were performed at a sweep rate of 0.23 V s^−1^ with a magnitude of 10 V. In order to record the *I*–*V* characteristics over a wide temperature range, the samples were transferred into a chamber with a Janis cryohead and measurements were carried out at 7.4 × 10^−5^ mbar in 10 K temperature steps between 260 and 370 K.

## 2 Results

**Fig. 1:**
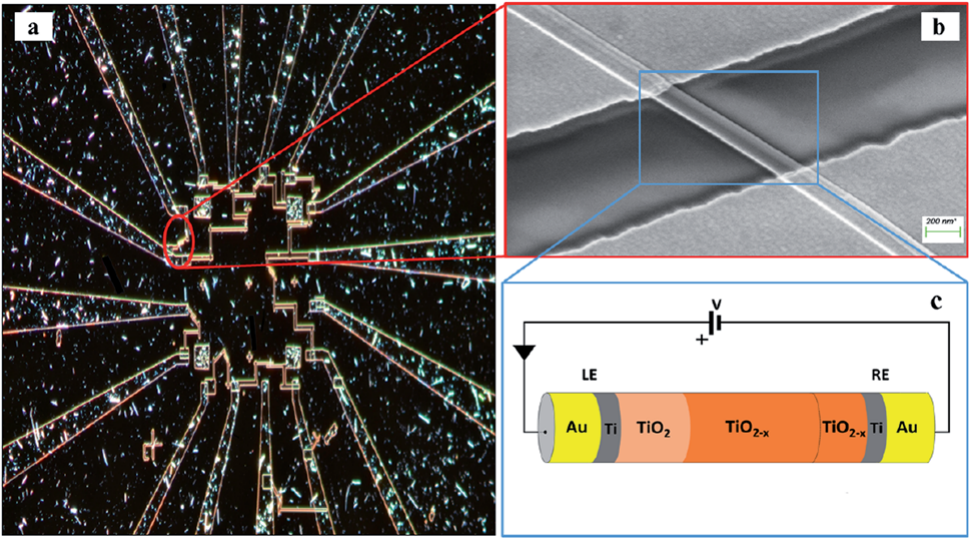
(a) Optical microscopy image of the Au–Ti/TiO_2_/Ti–Au device after three steps of dropcasting, EBL and metallization. (b) SEM image of the nanowire. (c) Schematic diagram of the device after applying a positive bias voltage *V* to the left electrode (LE). ‘RE’ stands for ‘right electrode’.

RRAM devices with the MIM structure Au–Ti/TiO_2_/Ti–Au were fabricated. Each device consisted of a single crystal TiO_2_ nanowire and two Ti/Au electrode contacts. Fig. 1(a) is the optical microscopy image of the Au–Ti/TiO_2_/Ti–Au device after three steps of dropcasting, EBL and metallization. Schematic and SEM images of the nanowire device are also shown in Fig. 1. Electrical characterization measurements were performed by initially electroforming the device in air followed by *I*–*V* sweeps measured under both air and vacuum conditions. The forming process was conducted with one of the electrodes held at ground and used as a reference throughout. A 5 V bias was applied on the other electrode for 10 minutes using a Keithley 4200 SCS^19,28^ and the current was monitored with a set compliance limit of 100 nA. This forming stage results in the creation of oxygen vacancies at the anode (positive electrode) which drift under the applied field towards the cathode supplementing the native concentration of vacancies present in the wire. Vacancy production is accompanied by local O_2_ production at the anode, which presumably dissolves into the electrode or escapes into the ambient environment. Some of this trapped O_2_ may also recombine with vacancies at the anode end of the wire and can influence the conduction response of the wire.

**Fig. 2:**
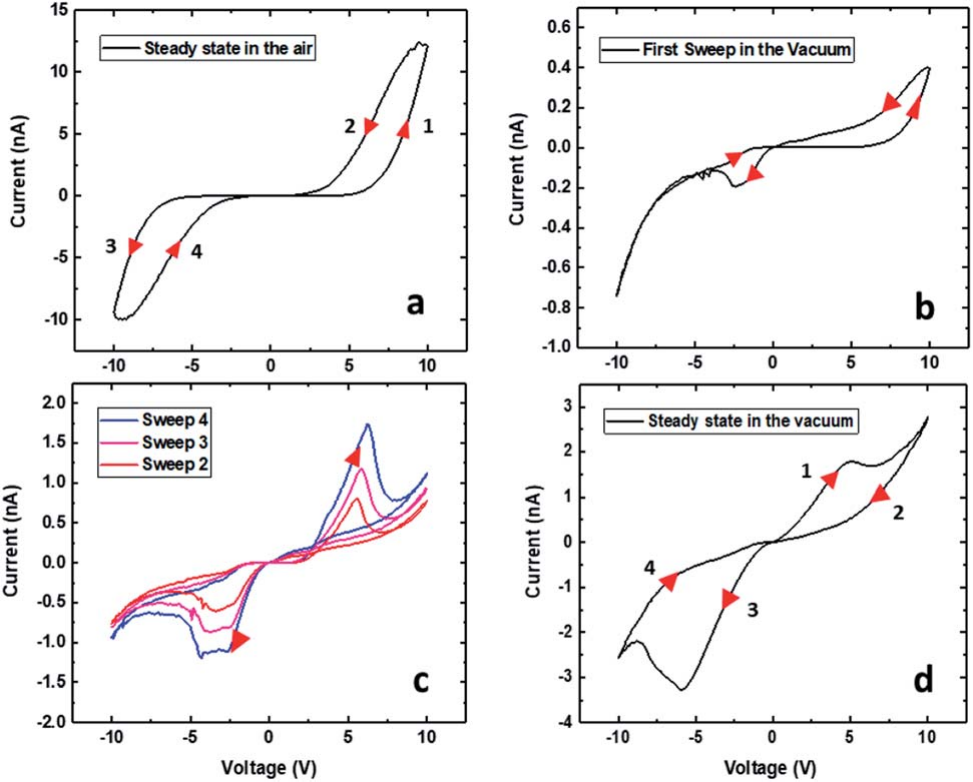
The *I*–*V* characteristics of an Au–Ti/TiO_2_/Ti–Au device in (a) air and under (b–d) vacuum measured for a full voltage sweep: (1) 0 → 10 V, (2) 10 → 0 V, (3) 0 → −10 V, and (4) −10 → 0 V. The arrows indicate the direction of the voltage sweep: measurements in air follow CCW switching whereas those under vacuum follow CW switching. (c) The *I*–*V* characteristics in a vacuum after 4 sweeps. (d) The steady state *I*–*V* curve under vacuum. All subsequent sweeps and the data were recorded at room temperature in all cases.

After successful forming steps in air, *I*–*V* measurements were performed at room temperature by sweeping the bias voltage applied to the device from 0 V → 10 V → 0 V → −10 V → 0 V. The data recorded in air in Fig. 2(a) are comprised of relatively featureless CCW hysteresis loops in both the forward and reverse bias directions with a gap in the current response around zero bias. Following air measurements, the device was transferred to a Janis chamber and pumped down to the 10^−6^ mbar range. The first *I*–*V* curve recorded in a vacuum is shown in Fig. 2(b) and is characterised by a CCW loop during the positive voltage sweep much like that found in air but with a lower current level. Remarkably, the loop observed during the negative voltage sweep in Fig. 2(b) is CW and shows clear evidence of emergent NDR behaviour. Subsequent sweeps in a vacuum (*cf.*Fig. 2(c)) show CW loops of increasing current amplitudes at both polarities, including the presence of growing NDR features and the elimination of the gap in the current response around zero bias. The steady-state *I*–*V* characteristics of the device in a vacuum at room temperature are shown in Fig. 2(d) in which we have highlighted four branches of the hysteresis loops labelled 1: 0 → 10 V, 2: 10 → 0 V, 3: 0 → −10 V, and 4: −10 → 0 V. By ‘steady state’ we mean that the whole *I*–*V* hysteresis is reproducible upon application of subsequent voltage sweeps. The forward hysteresis loop exhibits a NDR region in branch 1 in which current decreases when the voltage varies between +5 V and +7 V.^21^ The current then increases again when the voltage goes above +7 V up to a maximum value of +10 V. This NDR behaviour is also evident in the reverse voltage-scan between −6 V and −9 V (branch 3).

The strikingly different behaviour of the device in air and under vacuum, the opposite sense of the hysteresis loops, and the absence and presence of NDR behaviour indicate that our Au–Ti/TiO_2_/Ti–Au devices exhibit complex memristive phenomena beyond the simple ion-drift memristor picture^6^ commonly used to explain the conduction properties of TiO_2_-based systems. All TiO_2_-based devices have n-type semiconductor channels with mainly donor-type defects, including oxygen vacancies and titanium interstitials, which are responsible for the n-type conductivity, resulting from the oxygen deficient TiO_2−*x*_ stoichiometry.^29–31^ The existence of charged donors that can drift in response to an applied electric field is known to account for the memristive properties of TiO_2_ devices. In particular, there is a tendency of field-induced drift to result in the formation of a nonstoichiometric TiO_2−*x*_ layer with a high concentration of positively charged oxygen vacancies at the negatively biased electrode (cathode). In our Au–Ti/TiO_2_/Ti–Au device, the presence of the Ti adhesion layer is a source of additional complexity since under the appropriate bias conditions it can drive the electrochemical oxidation of TiO_2_ to create a locally high concentration of oxygen vacancies at the Ti/TiO_2_ interface of the positively biased electrode (anode). Other factors that can play a role in the transport response of our nanowires are the presence of surface defects; there are defects/traps along the length of the wire, and they can impact the device conductance as they become filled/emptied by interaction (or not) with the air. In fact, TiO_2_ is polycrystalline and disordered, hence other means of characterization such as X-ray photoelectron spectroscopy (XPS) would be required in order to estimate the oxygen concentrations and defect profile. However, we will mostly view the nanowire as a parallel conductive channel at best; defects do not pinch off the wire as a conductance channel. The Schottky barriers at the electrodes, on the other hand, will be the dominant transport mechanism as revealed by our models fitted to the experimental data.

In this paper, we will describe how the interplay between oxygen vacancy drift towards the cathode and oxygen vacancy production at the anode in the presence or absence of ambient oxygen can account for the observed device behaviour. At sufficiently low voltages in a vacuum, the forward loop (branch 1) in Fig. 2(d) shows increasingly current behaviour indicating the presence of sufficient oxygen vacancies along the wire channel to facilitate conduction so that the wire is in a low resistance state (LRS). This contrasts with the behaviour in air (*cf.*Fig. 2(a)) where recombination of oxygen vacancies with O_2_ in the ambient environment always results in a current gap in the low voltage range. This suggests that O_2_ recombination with oxygen vacancies is not important under vacuum conditions and the vacancies produced during device operation continue to facilitate conduction in the wire, ultimately reaching the steady state behaviour seen in Fig. 2(d). Nonetheless, the reduction in current in the NDR region (observed only when the system is in a vacuum) suggests that somehow the concentration of oxygen vacancies within the channel is disrupted. We attribute this behaviour to the formation of Schottky barriers^32–34^ at the interfaces of the device. During forward bias, the device channel close to the anode becomes depleted of oxygen vacancies resulting in Schottky barrier formation that can limit current flow through the channel. These NDR regions are volatile, however, and the *I*–*V* data in Fig. 2(d) reveal a second enhancement in current as the voltage increases beyond +7 V. We attribute this latter behaviour to the well-known electrochemical oxidation of TiO_2_ at the anode which results in the generation of additional oxygen vacancies that then drift along the wire towards the cathode. In order to verify the robustness of the NDR regions, we measured the *I*–*V* characteristics in a vacuum over a wide temperature range (260–370 K) in 10 K increments (*cf.* Fig. S7 in the ESI†). These temperature dependency studies reveal that the NDR regions are more pronounced at higher temperatures (above 300 K), consistent with the importance of thermally assisted diffusion of the charge vacancies along the device during the creation and subsequent elimination of the Schottky barrier.

In contrast to the behaviour in a vacuum, hysteresis loops in air show the presence of a large current gap until the applied voltage exceeds 5 V, indicating that oxygen vacancies produced during forming or cycling the device recombine with O_2_ in the ambient environment. As a result, the wire is non-ohmic and in a high resistance state (HRS) due to low oxygen vacancy concentration at low voltages (*cf.*Fig. 2(a)). When the applied bias exceeds 5 V, which corresponds to the characteristic voltage at which vacancy production commences at the anode, additional vacancies are injected into the wire. As the voltage increases further, the rate of vacancy production wins out over O_2_ recombination and the current increases accordingly. On the return trace, when the voltage drops below 5 V and approaches 0 V, vacancy production is turned off, and recombination depletes the oxygen vacancy level in the wire, resulting in a wide gap in the current response around zero bias. In this scenario, during the reverse bias loop, the wire is in essentially the same condition as at the start of the forward loop (*cf.*Fig. 2(a)), and for this reason, both switching directions will be CCW; the only difference is that the vacancy production will take place at the other end of the wire. Based on this analysis, we adopted extended nonlinear memristive descriptions^24–26^ that involve the competition between the Schottky barrier and ohmic-like contact dominated by tunnelling to describe the dynamics of the active ion transport channels that are formed within the wire during *I*–*V* sweeps. These approaches also allow for the inclusion of diffusion and retention terms^25,35^ to account for the non-uniform mobility and generation of oxygen vacancies inside the wire. The model equations can capture the dynamics of the ionic mechanisms and describe the complex *I*–*V* data obtained from our Au–Ti/TiO_2_/Ti–Au devices. This extended memristive picture is inspired by previous implementations that consider nonlinear doped channels formed at the Schottky interfaces in which transport is primarily regulated by the device contact resistances rather than the conductance of the channel.^34,36^ The interplay between Schottky and tunnelling transport offers new voltage threshold possibilities that can be applied in multilevel memristor memory cells for RRAM^37^ and is presented below.

### 2.1 Nonlinear ion-drift memristive model

The simplest memristive description is the linear ion drift model proposed by the HP Labs team^6^ in which the characteristic pinched *I*–*V* hysteresis of a memristor^38,39^ can be obtained using ohmic response combined with a state equation that describes the depletion layer drift with the amount of current flowing through the device. Although intuitive and capable of capturing the basic operation mechanisms of memristive behaviour, numerous experiments have demonstrated that memristive response can deviate considerably from the linear ion-drift picture. The complex *I*–*V* loops containing NDR regions and varying switching directions in our Au–Ti/TiO_2_/Ti–Au devices in a vacuum (*cf.*Fig. 2(d)) will be described using variances of the nonlinear ion-drift picture found in the literature,^11,40–42^ which assumes a nonlinear voltage-controlled dependency between the input voltage and an internal state variable, *x*. The latter is a weight ∈ [0, 1] that regulates the contribution of relevant transport regimes to the device conductance. Note that *x* should not be simply interpreted as a ‘filament length’ or a depletion layer divider as in the HP model with resistors in series. We will discuss its interpretation in more detail later on. The NDR region in the measured *I*–*V* curve in a vacuum suggests that our device operates similarly to a tunnel-diode, a two-terminal semiconductor element that also exhibits NDR. For this reason, we apply the nonlinear memristive model that assumes^24^ two main contributions for the current propagation, Schottky (diode-like) and ohmic-like contacts dominated by tunnelling current. The memristor equations for this model are given by

1 *I* = (1 − *x*)*I*_Schottky_ + *xI*_tunnelling_ = (1 − *x*)*α*[1 − e^(−*βV*)^] + *xγ* sinh(δ*V*)

**Fig. 3:**
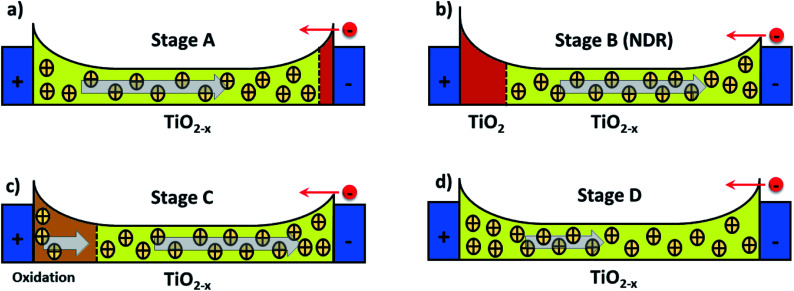
Schematics illustrating ionic events near the Schottky interfaces; these are viewed as the main charge transport mechanisms in our TiO_2_ nanowire devices under vacuum. The panels illustrate dopants (positive charges) and energy band profiles at four key stages during the positive voltage sweep shown in Fig. 2(d). Mobile oxygen vacancies in the TiO_2−*x*_ are the main entities that will regulate the height of the Schottky barriers at the contacts, enabling electrons (red circle) to leave the valence band to establish n-type current. The left and right blue rectangles represent the electrodes: the positive terminal is the anode whereas the negative terminal is the cathode. The vertical dashed lines delimit the regions of high (yellow/brown) and low (red) dopant concentrations. The arrows indicate the direction in which oxygen vacancies drift in response to the bias voltage. (a) Representation of dopants drifting towards the cathode in the initial low voltage range (*V*_bias_ ≲ +5 V), branch 1. Oxygen vacancies populate most of the nanowire and the device starts the voltage sweep at a highly conducting state. (b) A deficiency in oxygen vacancies at the anode occurs as ions continue to drift towards the cathode, causing the current drop at *V*_bias_ ≈ +5 V (NDR). (c) At *V*_bias_ ≈ +7 V, oxidation at the anode results in the generation of extra dopants that will drift through the channel. This causes the second current increase observed in branch 1 of the *I*–*V* curve. (d) As the bias voltage is brought back to zero at branch 2, the drift velocity of the dopants is gradually reduced as represented by the shorter arrow. Note that the effective length of the device may have increased due to the diffusion of oxygen vacancies to the pure Ti contact layers (not shown).

2
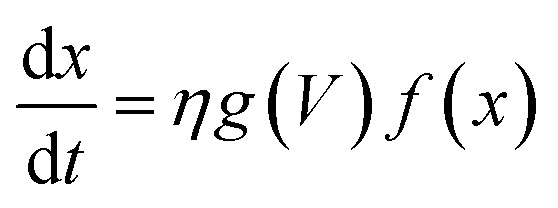
where the first term in eqn (1) is the Schottky barrier contribution at the interface between TiO_2_ and an electrode contact. The second term is the tunnelling contribution that can be dominant at the TiO_2−*x*_/electrode contact. *V* is the voltage input signal. These two contributions compete and are weighted by a normalized quantity, *x*, whose dynamics is governed by the equation of motion in (2). *η* = ±1 is the polarity coefficient and *g*(…) typically is a nonlinear monotonic function of the applied voltage *V* and it can also define the effects caused by threshold voltages in the memristive operation. *f*(…) is a window function used to account for nonlinearity effects at the device's boundaries. *α*, *β*, *γ*, and *δ* are positive parameters that can be determined by fitting eqn (1) and (2) onto experimental *I*–*V* curves. The question now is what functional form should we use in (2) to capture the highly complex shape of the *I*–*V* curve in Fig. 2(d) and its dual CW orientation? For that, we will first reflect on the transport mechanisms occurring inside the nanowire at each *I*–*V* branch, having in mind that the nonlinear movement of ions within the wire will play a pivotal role in the respective contributions coming from the Schottky and tunnel channels to the current. Fig. 3 shows in a schematic way the distribution of the oxygen vacancies within the wire – at room temperature and in a vacuum – in a positive bias voltage range, *i.e.* during branches 1 and 2 of Fig. 2(d). These branches can be decomposed into four stages and Fig. 3 details the particular charge transport mechanisms taking place close to the electrode contacts. Stage A is associated with the initial current increase (before the NDR) in which dopants drift in response to the bias voltage. The positive voltage loop is CW, indicating that the nanowire initiates the sweep in a highly conductive state with most of the oxygen vacancies populating the channel. As voltage increases and oxygen vacancies keep drifting towards the cathode, a deficiency of dopants can occur at the anode as illustrated in stage B. The height of the Schottky barrier at the anode increases causing the NDR phase observed in the *I*–*V* curve. At sufficiently high voltages, oxidation at the TiO_2_/anode interface triggers the generation of extra oxygen vacancies that will drift in response to the bias as pictured in stage C. This causes the second increase in current in branch 1 of the *I*–*V* curve. As the voltage is brought back to zero in branch 2, the drift velocity of the dopants will reduce (nonlinearly) as represented in stage D. The same picture can be extended to the negative voltage range. Note that the negative voltage loop orientation is also CW and this could only be achieved if the system state for *V* → 0^−^ is at a high concentration of dopants across the channel as represented in stage D.

To capture the rich mechanism presented in Fig. 3, we applied exponential ionic drift forms^24,43,44^ in eqn (2) which – coupled with eqn (1) – reproduces the experimental *I*–*V* curve shown in Fig. 2(d). Yet, a few considerations have to be made in the models in order to account for the following facts: (i) the overall dynamics relies on the contributions of two Schottky barriers that can be formed not only close to the cathode as normally considered (TiO_2_/metal contact) but also at the anode (TiO_2−*x*_/metal contact); (ii) the respective Schottky contacts are affected by the drift of ions and oxidation at the anode. Such an interplay at the interfaces is also responsible for the dual CW loop at positive and negative voltage sweeps which the model should be able to fit. We assume that these two Schottky barriers do not coexist (they appear at distinct times during the voltage sweep), and for this reason, a single *I*_Schottky_ term in eqn (1) is enough (for now) to model their recurrent contributions. Any other conductive channel opened within the wire (*e.g.* ohmic-like, direct tunnelling, or even residual/leak currents) is modelled in the *I*_tunnelling_ term, and *x*(*t*) should be able to weight over these two conducting channels. We define a first order memristive model (MM1) based on the description of Chang *et al.*^24^ in which the dynamics of the internal variable *x* is modelled as3
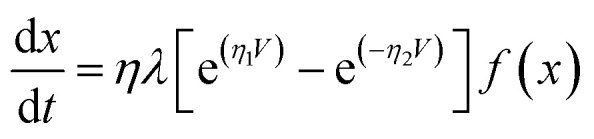
where *λ*, *η*_1_, and *η*_2_ are positive parameters determined from the fitting with the experimental *I*–*V* curve and we used the Joglekar window function^45^ in the majority of fittings. To test the robustness of our fitting procedures, we applied other functional forms to probe the dynamics of the state variable and other window functions.^46,47^ We achieved successful fitting results with the functional forms of Linares-Barranco *et al.*^43^ and Yakopcic *et al.*^44^ as well, confirming that the internal state variable is best described by exponential ionic drift models. Yet, our very first attempts to fit a memristive model onto our *I*–*V* curves were conducted with extended versions of the linear ion-drift equations proposed by the HP group^6^ in which we tried to devise a multilayer approach for the ionic drift with multiple fronts of doped layers^48–50^ being created and moving at distinct rates.^35^ This approach did not fit naturally onto our data as a result of the multiple nonlinearities our *I*–*V* curves exhibit which led us to try the nonlinear drift approaches.

The systems of eqn (1) and (2) form the basis of the nonlinear ion-drift model which will fit to the experimental *I*–*V* data shown in Fig. 2. For this, all parameters embedded in those equations will be optimized in accordance with the *I*–*V* experimental data points. This optimization is performed numerically using a nonlinear least-square minimization routine based on the Levenberg–Marquardt method with constraints.^27^ The optimization consists of minimizing the objective function given by the chi-square statistic 
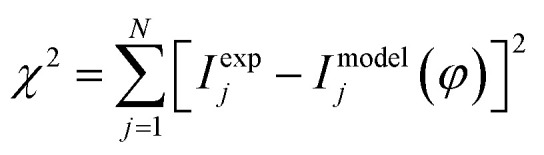
 in which *I*^model^ is the numerical outcome of eqn (1), *I*^exp^ is the measured current data, and *N* is the total number of data points. *ϕ* represents the set of parameters to be optimized in the fit, and for the case of eqn (1)–(3), it is given by *φ* ≡ {*α*, *β*, *γ*, *δ*, *λ*, *η*_1_, *η*_2_}. Constraints were imposed for all parameters, *e.g.* they all have to be positive and 0 < *x* < 1. Note that we are solving a nonlinear problem that is heavily dependent on the initial conditions. Nonlinear fittings are challenging, and the model can occasionally differ significantly from the experimental results if the initial parameters are not set sufficiently close to their optimal values. In fact, it is not uncommon to find cases in which the *I*–*V* curves are well fitted but the numerical solution for *x*(*t*) is not physically meaningful. In this way, our optimization strategy does not intend for the most perfect quantitative *I*–*V* fit; it aims at providing insight on the dynamics of the dopants and on the impact caused by the Schottky barriers at the contacts. More specifically, our goal is to gain qualitative understanding of the respective contributions of the main conducting channels (Schottky and direct tunnelling) of the device by extracting this information from the numerical fittings of the experimental *I*–*V* curves.

## 3 Discussion

### 3.1 Results under vacuum

**Fig. 4:**
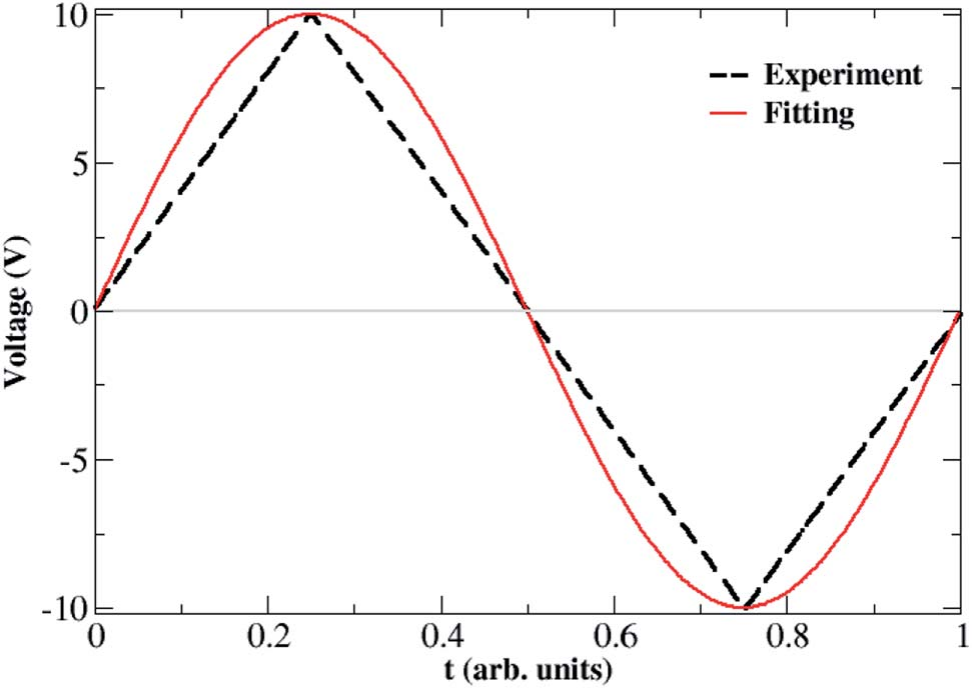
(Dashed line) Triangular waveform model of the input voltage used to simulate the experimental voltage sweeps shown in Fig. 2. (Full line) A sinusoidal waveform with *V*(*t*) = *V*_0_ sin(2π*t*) and *V*_0_ = 10 V outlining the original signal is used in the fitting procedures in order to avoid the piece-wise aspect of the triangular signal.

Before presenting the outcomes of the fittings, it is important to specify the form of the input signal, *V*(*t*), which will be part of the fitting functions. Fig. 4 shows the waveform model of the voltage input used to simulate the voltage sweeps in the experiments; it consists of a one period triangle waveform of voltage *versus* time with an amplitude of 10 V. The timeline is expressed in arbitrary units (a.u.) for the sake of simplicity. For the fitting, however, we used a sinusoidal waveform of *V*(*t*) = *V*_0_ sin(2π*t*) with *V*_0_ = 10 V (as shown in Fig. 4) in order to avoid the piece-wise aspect of triangular waveforms.^51^ Such a modification in the shape of the input signal does not impact remarkably our qualitative analysis and guarantees the continuity of the fitting procedure.

**Fig. 5:**
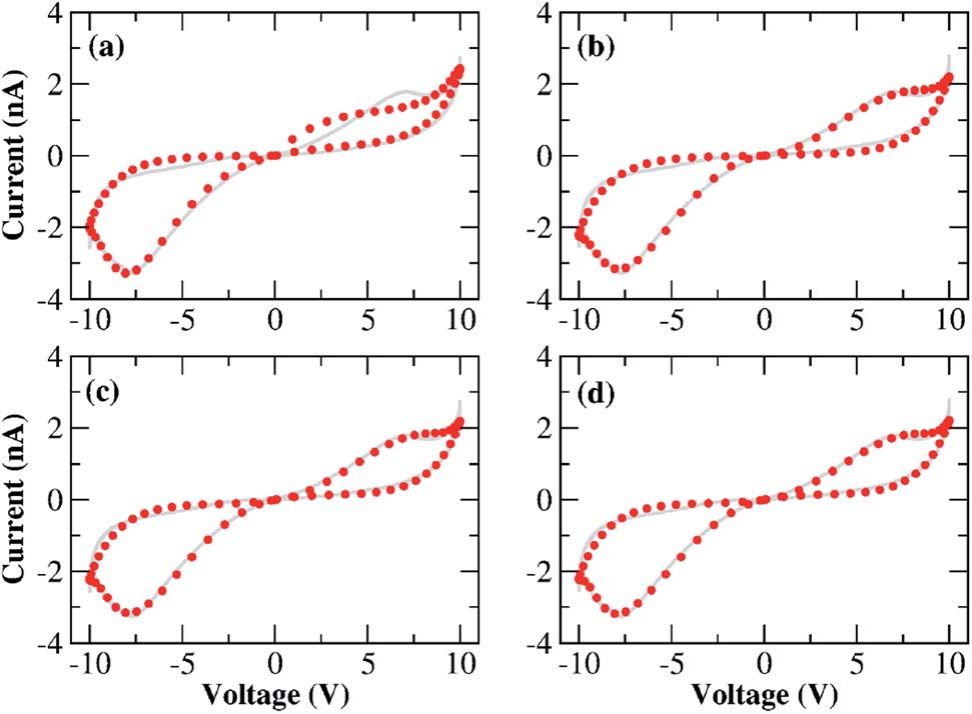
Results for the numerical fittings (circular symbols) of the *I*–*V* characteristics of our Au–Ti/TiO_2_/Ti–Au device in a vacuum using the four nonlinear memristive models (MM) discussed in the main text: (a) nonlinear ion-drift (MM1), (b) nonlinear ion-drift with static diffusion (MM1 + *τ*), (c) nonlinear ion-drift with dynamic diffusion (MM2), (d) nonlinear ion-drift with dynamic diffusion and retention (MM3). (Grey solid line) The same experimental *I*–*V* characteristics depicted in Fig. 2(d) but splined and projected onto the voltage input shown in Fig. 4. See the main text and ESI† for further details on the modelling scheme and on the resulting fitting parameters. Tables S1–S4 in the ESI† contain the optimized values of all parameters for all four nonlinear memristive models used to fit the *I*–*V* characteristics. All fittings follow the dual CW orientation of the *I*–*V* hysteresis.

Fig. 5 shows the result of the nonlinear least-square minimization conducted on the experimental *I*–*V* characteristics presented in Fig. 2(d) using four levels of the nonlinear ion-drift model which we will detail in the following. The values of all optimized parameters can be found in the ESI.† The first order memristive model (MM1) comprises the simplest ion-drift description using eqn (1) and (2) and its fitting is presented in panel (a). The model can reproduce most of the nonlinearities of the *I*–*V* curve but it fails significantly in the NDR region of branch 1. An attempt to improve this description, particularly in the NDR region, is to add a diffusion term in the state equation as4
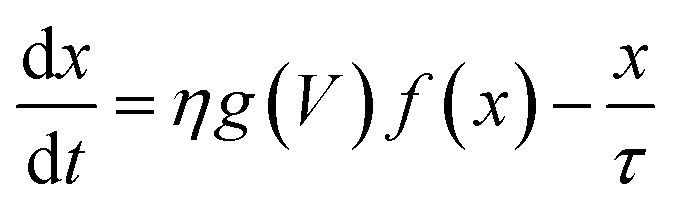
where *τ* is the diffusion time parameter that can also be determined through fitting.^24–26^ This model is referred to as ‘MM1 + *τ*’. The diffusion term is expected to hinder the current flow through the device and this may result in a better description of the NDR region. In the absence of a bias voltage, the diffusive contribution gives an internal state variable that decays as *x* = *x*_0_ e^−*t*/*τ*^ with *x*(*t* = 0) = *x*_0_. When diffusive contributions do not play a role, *τ* → *∞*. Fig. 5(b) shows the resulting fitting using a nonlinear ion-drift model with a diffusion term. The fitting is considerably improved overall and it yielded a diffusion rate of *τ* = 0.174 a.u.

A second order of refinement in the memristive model (MM2) was tested using a phenomenological picture proposed in ref. 26 and 35 which assumes the decay rate *τ* to be a variable affected by the electric field in the same way as *x*. *τ* is also referred to as a ‘forgetting’ rate^25^ meaning that the larger the *τ*, the more time the device needs to forget all its conductance history. Our experiments in a vacuum confirm that the characteristics of the system are volatile during the voltage sweep and this volatility could be simulated by considering the decay rate as a variable. MM1 + *τ* already demonstrated that diffusion can emulate the NDR effect caused by the deficiency of oxygen vacancies at the anode, but oxidation at the anode at higher voltages could impact the diffusion contribution. This supports the idea of a state equation ruling the dynamics of *τ*. MM2 couples eqn (1) and (2) with the following dynamic equation for *τ*,5
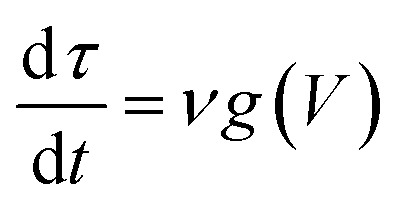
where *ν* and parameters inside *g*(*V*) are obtained from fitting. MM2 also fits well onto the data as shown in Fig. 5(c). MM2 is seen to improve particularly the fitting in branches 2 and 4. The numerical solution for *τ*(*t*) can be found in Fig. S2 in the ESI† and it shows how the diffusion rate is modulated over time. *τ* exhibits minima in the time ranges where the device reaches NDR stages, which is consistent with our interpretation of the ‘forgetting’ rate and how it is linked to the NDR regions.

A third order memristive model (MM3) proposed in ref. 26 and 35 was finally tested to fit our results and its outcome is shown in Fig. 5(d). MM3 adds another layer of complexity to the description which is the inclusion of a third dynamic variable which is the retention *ε* whose coupling with the state equation for *x* is given by6
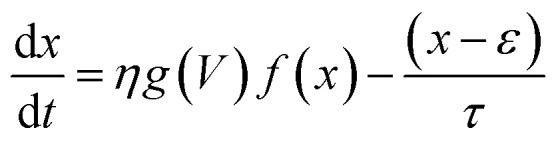
7
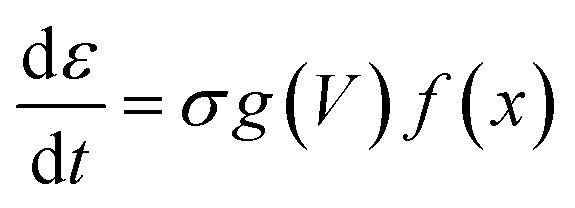
where *σ* and parameters inside the function form *g*(*V*) are obtained from fitting. Contrary to *τ* that accounts for the short-term plasticity of the device and volatility, the retention accounts for restoring the long-term memory of the device in response to redox reactions taking place inside the channel. Note that the diffusion term is related to the Fick diffusion law which occurs in response to concentration gradients within the device.^10,52^ If enough oxygen vacancies are able to bridge the two electrodes, we can say that a conductive channel for electronic propagation was formed. But in a scenario schematized as in Fig. 3, a conductive channel can be disrupted as a result of scarceness of oxygen vacancies particularly close to the device interfaces. Hence, the effect of diffusion is to decrease the conductance of the device by simulating the diffusion of oxygen vacancies out of the active conductive channel. The retention term is related to the Soret diffusion,^52^ a mechanism that accounts for the diffusion of particles in response to temperature gradients. The latter favours conductivity by attracting more oxygen vacancies to the active conductive channel which tends to have locally higher temperatures due to Joule heating. In our transport picture in Fig. 3, this term can be relevant to simulating the generation of extra oxygen vacancies at the anode which results in the current kick-in after the NDR regions. This model is also capable of modulating the experimental *I*–*V* curve but its fitting does not differ significantly from that of the MM2. The addition of another degree of freedom (*ε*) to the model and the fact that the fitting is not significantly perturbed demonstrate the robustness of the nonlinear ion drift-diffusion equations in describing the *I*–*V* curve of our Au–Ti/TiO_2_/Ti–Au device in a vacuum.

Tables S1–S4 in the ESI† present the values of the fitting parameters of all four memristive models used to fit the *I*–*V* characteristics in Fig. 2(d). Some of these parameters can be used to extract information about the Schottky barrier as discussed in Section 1 of the ESI.† Further analysis of the *I*–*V* hysteresis curves is also provided in the ESI† in which we split the *I*–*V* loops into Schottky, *I*_1_(*t*) = [1 − *x*(*t*)]*I*_Schottky_(*t*), and tunnelling, *I*_2_(*t*) = *x*(*t*)*I*_tunnelling_(*t*), contributions (*cf.* Fig. S1 in the ESI†).

**Fig. 6:**
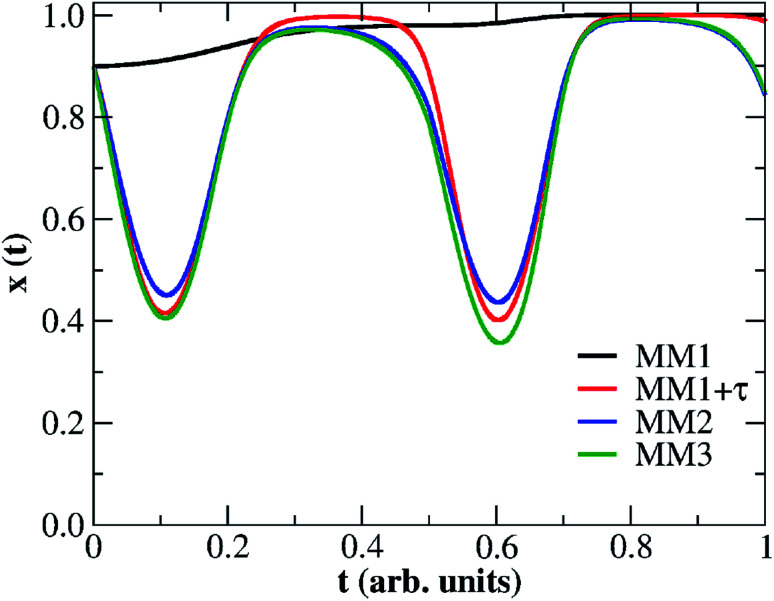
Time evolution for the normalized thickness of the conductive channel, *x*, at the device interfaces obtained from fitting four nonlinear memristive models: (black line) pure ion-drift (MM1), (red full line) ion-drift with a static diffusion term, *i.e. τ* = constant (MM1 + *τ*), (green line) ion-drift with a dynamic diffusion term, *i.e.* d*τ*/d*t* = *f*(*t*) (MM2), (blue line) ion-drift with dynamic diffusion and retention terms, *i.e.* d*τ*/d*t* = *f*(*t*) and d*ε*/d*t* = *h*(*t*) (MM3). *f*(*t*) and *h*(*t*) represent time-dependent functions.

Fig. 6 depicts the solutions of the inner state variable *x*(*t*) calculated from the fittings of all four nonlinear memristive models. Solutions for the other dynamic quantities {*τ*(*t*), *ε*(*t*)} are presented in Fig. S2 and S3 in the ESI.† Note that our criteria for best fitting not only include the best quantitative *I*–*V* matching, but also provide a meaningful physical outcome for the internal state variables. We tested numerous initial conditions for the fitting parameters including distinct values for *x*(*t* = 0) = *x*_0_. The best fitting outcomes resulted from 0.7 ≥ *x*_0_ ≥ 0.9 which gives a higher initial weight for the tunnelling current than for the Schottky current. The MM1 is ruled simply by drift and the dynamics of *x*(*t*) is set with voltage thresholds that account for the dual CW orientation of the *I*–*V* curve. The creation of extra oxygen vacancies at the anode in the forward voltage is responsible for restoring transport channels at the anode, enabling the device to start the reverse voltage loop in a highly conductive state. The fact that *x*(*t*) increases nonlinearly over the whole time range is to be interpreted as tunnelling channels being reinforced. The poor fitting of MM1 onto the *I*–*V* curve yields a *x*(*t*) solution that exhibits plateaus when it tries to fit the NDR regions. The refined memristive models capture a reduction in *x*(*t*) which is seemingly relevant to simulate the NDR behaviour. As modelled with eqn (1), *x*(*t*) regulates the *I*_tunnelling_ and *I*_Schottky_ current contributions. When *x*(*t*) decreases, it reflects on a weight increase for the Schottky contribution which corroborates with our transport mechanism depicted in Fig. 3. Note also that the time evolution of the internal state variable in MM2 and MM3 finds *x*(*t* = 0) ≈ *x*(*t* = 1). Measurements of multiple voltage sweeps applied after the system reached the steady state *I*–*V* hysteresis show nearly identical loops, and therefore one expects the initial conditions of the device to be recovered after each voltage cycle.

**Fig. 7:**
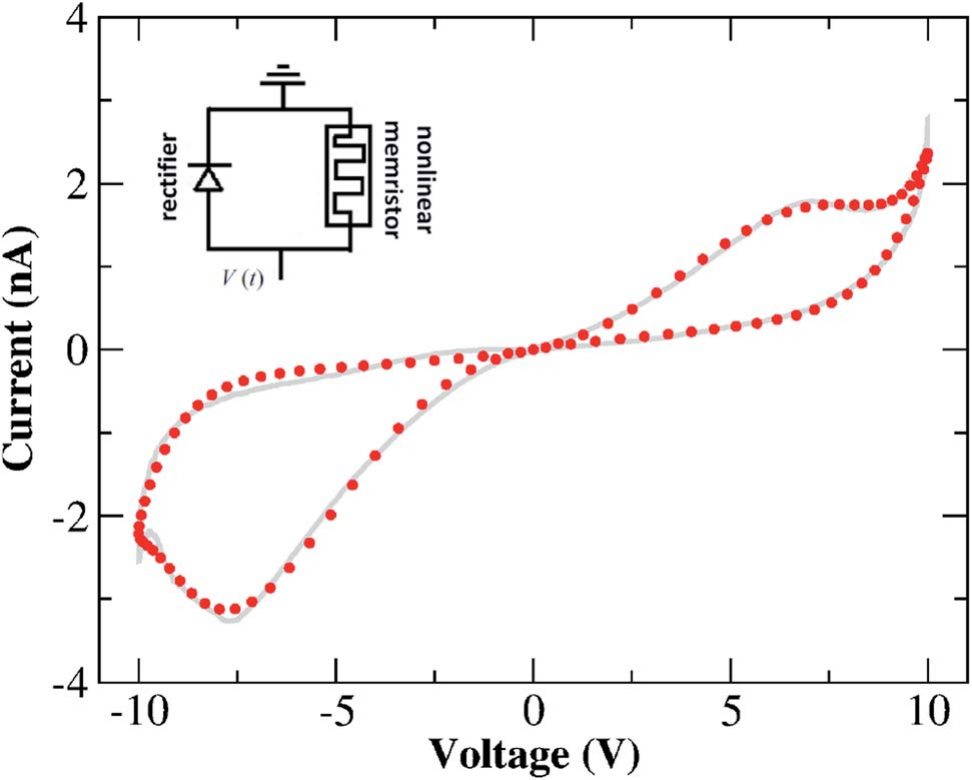
Result for the numerical fitting (circular symbols) of the *I*–*V* characteristics of our Au–Ti/TiO_2_/Ti–Au device in a vacuum using the response function (8) with the dynamic variable being described by the nonlinear ion-drift with static diffusion (MM1 + *τ*). (Grey solid line) The same experimental *I*–*V* characteristics depicted in Fig. 2(d) but splined and projected onto the voltage input shown in Fig. 4. Table S5 in the ESI† contains the optimized values of all parameters used to fit the *I*–*V* characteristics. The fitting follows the dual CW orientation of the *I*–*V* hysteresis. Inset: the equivalent circuit diagram for the memristive model in eqn (8).

One last ingredient was added to our memristive descriptions; in particular, this last consideration has the objective of trying to accentuate the fitting along the NDR regions of the *I*–*V* curve. As previously mentioned, the two Schottky barriers that dominate the transport response of the device are modelled effectively by means of a single Schottky current contribution as expressed in eqn (1). We will now decompose the Schottky contribution in eqn (1) as8
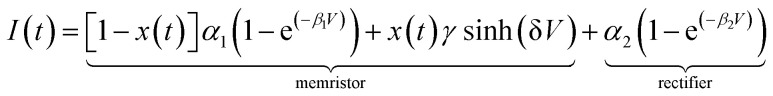
which considers the total current flowing through the device as the result of three parallel current channels, a Schottky (1st term) and a tunnelling (2nd term) channel regulated by the internal variable *x*(*t*), and a static rectifier channel (3rd term) to account for thermionic effects. This model aligns with the description proposed by Yang *et al.*^18^ in which an equivalent circuit composed of a memristor plus a rectifier was able to reproduce complex *I*–*V* hysteresis obtained in TiO_2−*x*_-based devices. In our case, the memristor is the joint contribution of the 1st and 2nd terms in expression (8) and the 3rd term is the rectifier characterizing thermionic contributions. The equivalent circuit for this model is shown in the inset of Fig. 7. The latter depicts the fitting that resulted from the response function (8) with the internal variable being described by MM1 + *τ*. The behaviour of *x*(*t*) obtained from this fitting (not shown) is similar to the modulation our higher order memristive models calculated as shown in Fig. 6. The fitting of the NDR region (especially the more evident one at *V* > 0) is considerably improved when Schottky and rectifying contributions are decomposed in the current response function. This agrees with the ionic mechanism illustrated in Fig. 3 in which the drift of ions towards the cathode exposes (partially) the pristine rectifying aspect of the device, and hence this contribution is expected to play a role. A sample code that reconstructs the fitted *I*–*V* curve in Fig. 7 with a set of optimized parameters is provided in the ESI.† Initial conditions and parameter variability tests were conducted for this improved memristive model and their outcomes are shown in Fig. S4 and S5 in the ESI.†

### 3.2 Testing the diffusive model

**Fig. 8:**
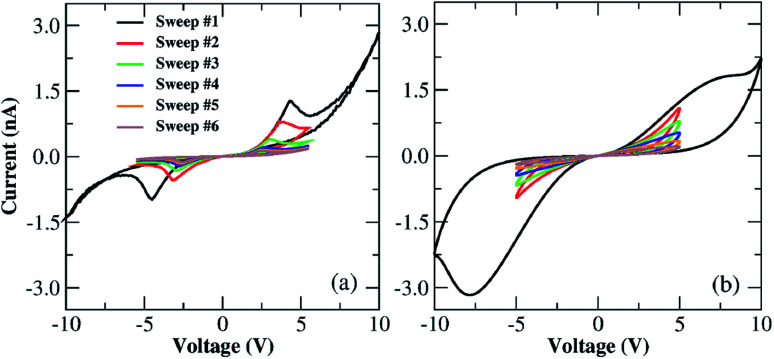
(a) Sequence of *I*–*V* characteristics measured for the Au–Ti/TiO_2_/Ti–Au device in a vacuum and at room temperature. A sequence of six voltage sweeps was carried out, the very first one being conducted within the voltage window of −10 to +10 V and the follow-up sweeps within −5 to +5 V. (b) Calculated sequential *I*–*V* loops using the parameterized MM1 + *τ* description. The very first *I*–*V* sweep is a repetition of the fitting in Fig. 5(b), followed by the numerical results obtained using the MM1 + *τ* equations subjected to a sinusoidal voltage signal of 5 cycles and an amplitude of 5 V. All loops follow dual CW orientation.

We conducted an extra measurement to evaluate the importance of considering diffusion effects in our Au–Ti/TiO_2_/Ti–Au wire devices. *I*–*V* characteristics were measured for 6 voltage cycles; for the first sweep the voltage amplitude is *V*_0_ = 10 V and for all subsequent sweeps, *V*_0_ = 5 V. This means that extra oxygen vacancies will be generated in the first sweep but not in subsequent ones since a maximum applied voltage of |5| V is not enough to activate the oxidation at the anode. This voltage threshold seems to be related to the minimum potential required to oxidize TiO_2_ to form new oxygen vacancies. A narrower voltage scan should not reveal any major current increase in the *I*–*V* loops since electrochemical generation of vacancies is turned off. The result of these measurements is shown in Fig. 8(a); the first sweep depicts the characteristic *I*–*V* butterfly shape with nearly symmetric NDR regions in the forward and reverse bias scans, followed by a nonlinear current dependence associated with the creation of secondary dopant concentrations within the channel. Subsequent sweeps show a continuous drop in the current response after each cycle. The robustness of this ‘dying out’ effect of the current is verified in another set of measurements presented in Fig. S6 in the ESI.† The device is in a vacuum, and surprisingly, even in the absence of recombination with O_2_ from the ambient environment, the vacancy concentration in the wire seems to decay slowly under vacuum. The absence of a current kick-in in these measurements means that extra dopants are no longer being formed by electrochemical oxidation.

We used MM1 + *τ* to recreate the multiple voltage sweep measurements shown in Fig. 8(a). For the sake of simplicity, an all-point nonlinear fitting was not conducted in this study. Instead, we used the already parameterized MM1 + *τ* to replicate the first voltage sweep as in Fig. 5(b), and for the follow-up sweeps, we tuned a few parameters manually to identify the conditions in which loop decays could be visualized. The result of this qualitative analysis is presented in Fig. 8(b). One can observe the *I*–*V* loops gradually dying out after each voltage sweep due to the diffusion contribution that degrades the weight of the tunnelling channel. Ion diffusion is often used to capture the ‘forgetting’ behaviour and short-term memory in memristive systems.^25^ With the gradual deterioration of the tunnelling contribution due to ion diffusion, the system “forgets” part of its drift history and the neighbouring loops fail to overlap because the tunnelling current is reduced after each voltage sweep. This result also confirms that ion-diffusion is an important mechanism in the conduction response of our Au–Ti/TiO_2_/Ti–Au wires since this narrower window voltage measurement screens its influence by not allowing electrochemical reduction to occur at the interfaces of the device.

### 3.3 Results in air

**Fig. 9:**
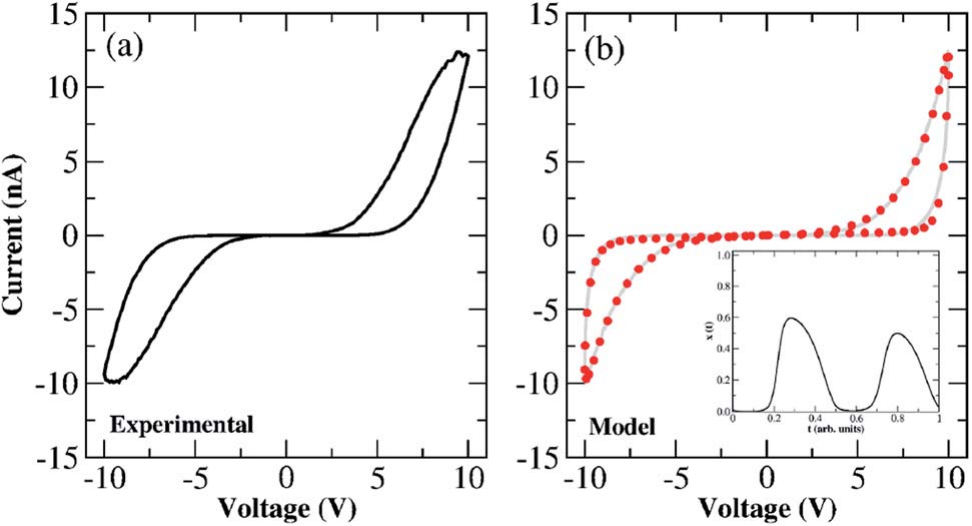
(a) *I*–*V* characteristics of the Au–Ti/TiO_2_/Ti–Au device measured for a full voltage sweep of an amplitude of 10 V exposed to air and at room temperature. Both forward and backward voltage scans follow CCW switching. These *I*–*V* characteristics are reproducible on subsequent sweeps. (b) (red circles) Fitted *I*–*V* loop using the MM1 + *τ* description exposed to a sinusoidal voltage waveform of an amplitude of 10 V. (grey line) Experimental current values depicted in panel (a) projected to the sinusoidal voltage waveform shown in Fig. 4. The fitting follows the dual CCW orientation of the experimental *I*–*V* hysteresis. (Inset) Temporal evolution of the internal state variable *x*(*t*), describing the weight of the tunnelling channel, resulted from fitting the MM1 + *τ* description onto the *I*–*V* experimental curve in panel (b). Values of the optimized parameters can be found in Table S6 in the ESI.†

To support all memristive models used to describe the dynamics of the tunnelling and Schottky channels within the Au–Ti/TiO_2_/Ti–Au device, we exposed the system to air from which we expect to isolate other aspects that corroborate with the ion drift-diffusion picture with recurrent concentration of dopants. Electrical characterization and modelling were also performed on the device at room temperature and under air conditions. Under these conditions, we observe a competition between the rate of vacancy production at the anode and vacancy recombination with the O_2_ from the ambient environment. This is evidenced in Fig. 2(a) (reproduced in Fig. 9(a)) which shows the experimental characterization of the device over a voltage cycle of an amplitude of 10 V. Fig. 9(b) depicts the fitting of MM1 + *τ* description onto the experimental *I*–*V* curve. It is worth remembering that the fitting was done using a sinusoidal voltage waveform rather than a triangular waveform (*cf.*Fig. 4) to avoid the piece-wise aspect of the input signal during the numerical optimization. This of course changes the shape of the *I*–*V* curve used in the fitting (*cf.*Fig. 9(b)) with respect to the originally measured one (*cf.*Fig. 9(a)). Overall, this alteration in the voltage waveform does not impact our results and conclusions qualitatively. In contrast to the behaviour in a vacuum, the hysteresis loops in air are CCW. The experimental data also show the presence of a large current gap until the applied voltage exceeds ∼|5| V, indicating that oxygen vacancies produced during the forming step are effectively recombined with the O_2_ in the environment. Oxygen deficiencies are known to lower the height of the Schottky barrier at the contacts, favouring the propagation of current through the device. The low levels of oxygen vacancy concentration at low voltages indicate that the Schottky barrier may be the main limitation to electron conduction. The current increases nonlinearly above |5| V which corresponds to the characteristic voltage in which vacancy production commences at the anode and additional vacancies are injected into the wire. As voltage increases, the rate of vacancy production wins out over O_2_ recombination and the current continues to increase accordingly. On the return trace, when the voltage drops below |5| V and sweeps towards 0 V, vacancy production is turned off, and recombination depletes the oxygen vacancy level in the wire, resulting in a wide gap in the current response around zero bias. In this scenario, during the reverse bias loop, the wire is in essentially the same condition as at the start of the forward loop, and for this reason, both switching directions will again be CCW; the only difference is that the vacancy production will take place at the other end of the wire. The solution for *x*(*t*) resulting from this fitting is shown in the inset of Fig. 9(b); it shows that, around zero voltage, the dominant transport contribution is Schottky as *x* ≪ 1. The optimized diffusion rate was calculated as *τ* = 0.021 a.u. which is considerably smaller than the values found for the device in a vacuum. This shows that diffusion plays a more significant role in impacting the tunnelling channel when modelling the Au–Ti/TiO_2_/Ti–Au devices in air. The fitting predicts successfully that the weight modulating the tunnelling contribution has to increase above the voltage thresholds where oxygen vacancy generation wins over O_2_ recombination. The behaviour of *x*(*t*) in the inset of Fig. 9(b) allows us to obtain a more concrete interpretation of its meaning; *x*(*t*) can reflect the concentration of mobile vacancies (*n*_sc_) in the proximity of the Schottky barrier,^34^*i.e.* one can associate the variable controlling the thickness of the conducting channel with the concentration of mobile vacancies in such a way that *x*(*t*) ↔ *n*_sc_(*t*). When *x* → 0, *n*_sc_ → 0 meaning that in one (or both) of the device interfaces, there is a low concentration of mobile vacancies. The barrier height is sufficiently high in this case resulting in the current gap in the *I*–*V* curve. The increase of *x*(*t*) as the voltage sweep progresses can be related to an increase in the concentration of mobile vacancies, *n*_sc_, at the interfaces, lowering its barrier height and hence favouring tunnelling. This interpretation aligns with other memristive pictures that investigate electro-migration phenomena with a state equation for the mobile vacancy concentration coupled with field emission equations describing current density in Schottky barriers.^34,36,53^

## 4 Conclusions

In this study, we report detailed transport characterization of a metal–insulator–metal device composed of an Au–Ti/TiO_2_/Ti–Au structure probed in distinct pressure environments and temperatures. TiO_2_ based devices are known to exhibit memristive response translated into *I*–*V* hysteresis loops which can be illustrated by means of ion-drift models of a depletion layer drifting in response to an input signal. Nonetheless, the addition of Ti at the device interfaces has unveiled additional physical phenomena – hitherto unexplored – due to the formation of Schottky barriers at the contacts. These significantly impact the shape of the *I*–*V* curves which do not exhibit a regular 8-shaped trajectory but a rather complex *I*–*V* response containing NDR regions, distinct switching orientations, and rectifying behaviour depending on the ambient conditions the device experiences. At room temperature and under vacuum conditions, the device depicts bipolar NDR regions and nonlinear CW switching behaviour, whereas in air, the NDR is replaced by bipolar rectifying behaviour with nonlinear CCW switching behaviour. This rich transport outcome calls for nonlinear memristive pictures that go beyond linear ion-drift models which are commonly used to elucidate memristive phenomena in TiO_2_ based systems. This variety in transport outcomes also calls for a description flexible enough to capture all these intricate features without losing the intuitive aspect that made such linear approaches highly applicable. In this way, we reveal all aspects of fitting *I*–*V* characteristics with high order nonlinear memristive descriptions that follow exponential ionic drift dynamics with diffusion and retention terms. These terms are used to emulate short- and long-term plasticity elements in memristive systems. The incorporation of diffusion/retention terms successfully accounted for the scarceness and recombination/generation of oxygen vacancies at the device contacts. These are essential mechanisms to explain the peculiar memristive dynamics appearing in different parts of the *I*–*V* hysteresis loops. Such rich memristive dynamics could pave the way for an enhanced memory capability in multi-bit memristive devices^54^ in which more information can be encoded in a spectrum of resistance states resolved from a single nanowire. This study also offers the potential to integrate memory and logic functions on the same device with the design of threshold logic circuits that operate based on NDR properties such as in resonant tunnelling diodes.

## Author contributions

S. A. performed the experiments and electrical characterization on the nanowires and co-wrote the paper. C. G. R. co-wrote the paper and, along with K. E. and M. S. F., developed the computational model and ran the simulations. C. G. R. led the computational efforts. J. J. B. led the experimental efforts and co-wrote the paper. All authors discussed and commented on the manuscript and on the results.

## Conflicts of interest

There are no conflicts to declare.

## Supplementary Material

NA-002-D0NA00195C-s001

NA-002-D0NA00195C-s002
