# Supplementary material for: Nonlinear ion drift-diffusion memristance description of TiO2 RRAM devices
Source: Nanoscale Adv. 2020 Apr 21;2(6):2514–24. doi: 10.1039/d0na00195c (PMC9419089; doi:10.1039/d0na00195c)
Supplement: NA-002-D0NA00195C-s001 [file NA-002-D0NA00195C-s001.pdf]

Electronic Supplementary Material (ESI) for Nanoscale Advances.
This journal is © The Royal Society of Chemistry 2020

Supplemental Material: Nonlinear Ion Drift-Diffusion Memristance Description of TiO_2 RRAM Devices

S. Alialy, et al.

1. Fitting parameters for nonlinear ion drift-diffusion models

Tables below present the values of the optimized parameters resulted from the numerical fittings of Figure 5 in the main manuscript. Our selected fitting shown in Figure 5(a) in the main manuscript (MM1) was obtained using the functional form for the voltage-controlled element in the drift equation dx/dt [1] as

$$\frac{dx}{dt} = sgn(V)\,\lambda\left(e^{\eta_1 V} - e^{-\eta_2 V}\right)f(x) \qquad (S1)$$

and Joglekar window function [2]. The $sgn(\ldots)$ function is added to account for the dual CW orientation of the I-V curve. The initial guess and optimized values for this model are shown on Table S1. The determination of the parameters initial guess was performed using a systematic "pre-search" technique in which a subset of initial parameters is kept fixed while others are allowed to fluctuate under certain constraints.

Table S1: Initial guess and optimized parameters for the MM1 discussed in the main text using equations (1+S1) to describe the nonlinear ion-drift dynamics. The resulting fitting is depicted on Figure 5(a) in the main manuscript which shows the I-V characteristics of the Au-Ti/TiO_2/Ti-Au device in vacuum and at room temperature. The initial condition for the internal state variable was set at $x(t = 0) = 0.9$.

Fitting Parameters	Initial guess	Optimized
α	13.34519	14.3441598
β	0.1078093	0.40016776
γ	9.068×10^{-5}	0.00442768
δ	1.039012	0.67310141
λ	3.254168	0.15850210
η_1	0.26103	0.23135252
η_2	0.3249405	0.43469131

Our selected fitting shown in Figure 5(b) in the main manuscript (MM1+τ) was obtained using the functional form for the voltage-controlled element in the drift equation dx/dt [3] as

$$\frac{dx}{dt} = \begin{cases} \lambda^{+}\left(e^{\eta_1 V} - e^{\eta_2}\right) - \frac{x}{\tau} & V > 0 \\ 0 & V = 0 \\ \lambda^{-}\left(e^{-\eta_3 V} - e^{\eta_4}\right) - \frac{x}{\tau} & V < 0 \end{cases} \qquad (S2)$$

and Joglekar window function [2]. This functional form is flexible in terms of establishing the correct voltage thresholds for the model and accounts for the dual CW orientation of the I-V curve. The initial guess and optimized values for this model are shown on Table S2.

Table S2: Initial guess and optimized parameters for the MM1+τ discussed in the main text using equations (1+S2) to describe the nonlinear ion-drift-diffusion dynamics. The resulting fitting is depicted on Figure 5(b) in the main manuscript which shows the I-V characteristics of the Au-Ti/TiO_2/Ti-Au device in vacuum and at room temperature. The initial condition for the internal state variable was set at $x(t = 0) = 0.9$.

Fitting Parameters	Initial guess	Optimized
α	8.345194	6.20570855
β	0.1078093	0.08420589
γ	0.005	0.00867033
δ	1.039012	0.62012385
λ^+	3.254168	4.52025108
λ^-	3.254168	2.86780854
η_1	0.26103	0.17219533
η_2	0.3249405	0.86523992
η_3	0.26103	0.22444130
η_4	0.3249405	1.11771695
τ	0.03998672	0.17401350

Figure S1 depicts the decomposed contributions of $I_1(t) = (1 - x(t))I_{schottky}(t)$ and $I_2(t) = x(t)I_{tunnelling}(t)$ (cf. equation (1) in the main text) for the conditions established on Table S2. Their arithmetical addition results in the I-V curve of Figure 5(b) in the main manuscript. Their separate contributions reveal the main transport mechanisms across the voltage sweep; it is mostly dominated by the Schottky functional form, modulated by the $(1 - x(t))$ solution, up to $\sim|7|$ V, when the tunnelling functional form, modulated by the $x(t)$ solution, is significant in the current kick-in region and the backward voltage sweep. Similar features were observed for the higher order memristive models MM2 and MM3.

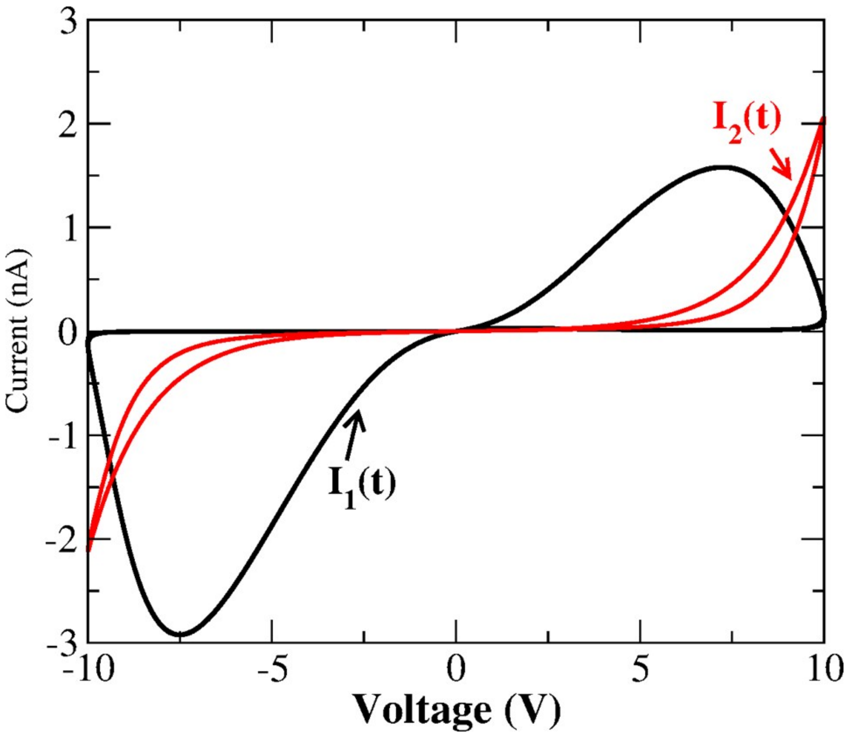

Figure S1: Calculated $I_1(t) = (1 - x(t))I_{schottky}(t)$ and $I_2(t) = x(t)I_{tunnelling}(t)$ decomposed contributions of equation (1) in the main text for the conditions established on Table S2. Their arithmetical addition results in the I-V curve of Figure 5(b) in the main manuscript.

Our selected fitting shown in Figure 5(c) in the main manuscript (MM2) was obtained using the functional form for the voltage-controlled element in the drift equation dx/dt [3] as expressed in equation (S2) and for $d\tau/dt$ [4] as

$$\frac{d\tau}{dt} = \begin{cases} \nu\left(e^{\eta_1 V} - e^{\eta_2}\right) & V > 0 \\ 0 & V = 0 \\ \nu\left(e^{-\eta_3 V} - e^{\eta_4}\right) & V < 0 \end{cases} \qquad (S3)$$

The initial guess and optimized values for this model are shown on Table S3. The optimized solution for $\tau(t)$ is depicted in Figure S2. When τ increases (decreases) points to a decrease (increase) in the diffusion effects within the device. Its dynamical behaviour agrees with our transport picture schematized in Figure 3 in the main manuscript.

Table S3: Initial guess and optimized parameters for the MM2 discussed in the main text using equations (1+S2+S3) to describe the nonlinear ion-drift-diffusion dynamics. The resulting fitting is depicted on Figure 5(c) in the main manuscript which shows the I-V characteristics of the Au-Ti/TiO_2/Ti-Au device in vacuum and at room temperature. The initial conditions for the dynamical quantities were set at $x(t = 0) = 0.9$ for the internal state variable and $\tau(t = 0) = 0.3$ a.u. for the diffusion rate.

**Fitting Parameters**	**Initial guess**	**Optimized**
α	6.8161	6.58505436
β	0.082169	0.08398121
γ	0.00823	0.00728259

δ	0.628513	0.63529386
λ^+	4.584937	3.98017841
λ^-	2.876534	2.58346814
η_1	0.168517	0.16214553
η_2	0.8989	0.78405950
η_3	0.227354	0.21776995
η_4	1.199935	1.26395065
ν	1.13166	2.26715151

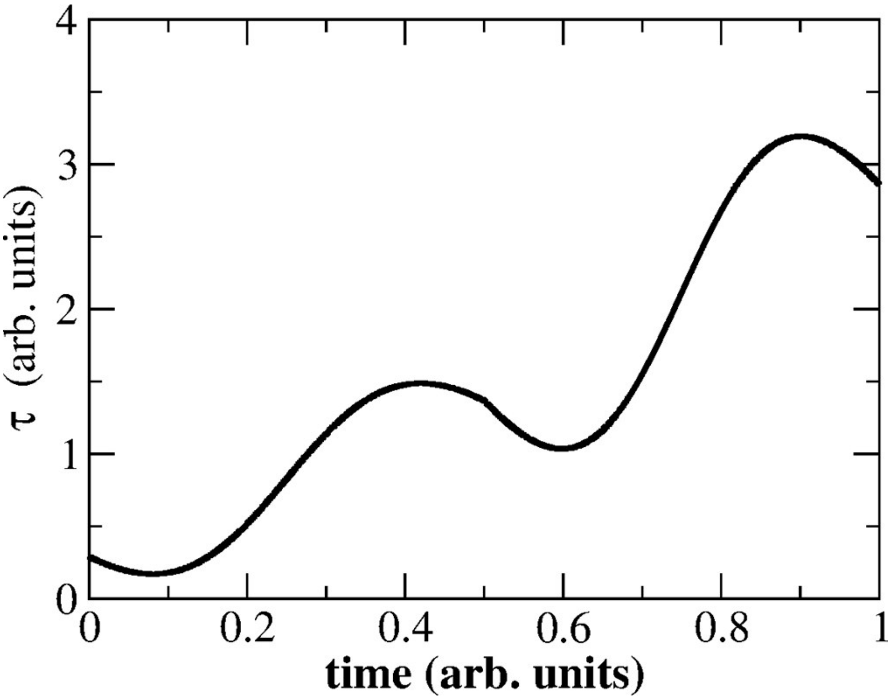

Figure S2: Calculated $\tau(t)$ (cf. equation (S3)) using MM2 with the conditions established on Table S3. This solution was obtained from fitting MM2 onto the I-V characteristics of Au-Ti/TiO_2/Ti-Au devices in vacuum and at room temperature (cf. Figure 5(c) in the main manuscript).

Our selected fitting shown in Figure 5(d) in the main manuscript (MM3) was obtained using the functional form for the voltage-controlled element in the drift equation dx/dt [3] expressed as

$$\frac{dx}{dt} = \begin{cases} \lambda^+\left(e^{\eta_1 V} - e^{\eta_2}\right) - \frac{(x-\varepsilon)}{\tau} & V > 0 \\ 0 & V = 0 \\ \lambda^-\left(e^{-\eta_3 V} - e^{\eta_4}\right) - \frac{(x-\varepsilon)}{\tau} & V < 0 \end{cases} \qquad (S4)$$

$d\tau/dt$ expressed as in equation (S3), and $d\varepsilon/dt$ [4] is given by

$$\frac{d\varepsilon}{dt} = \begin{cases} \sigma\left(e^{\eta_1 V} - e^{\eta_2}\right) f(x) & V > 0 \\ 0 & V = 0 \\ \sigma\left(e^{-\eta_3 V} - e^{\eta_4}\right) f(x) & V < 0 \end{cases} \qquad (S5)$$

with the Joglekar window function [2]. The initial guess and optimized values for this model are shown on Table S4. The optimized solutions for $\{\tau(t),\varepsilon(t)\}$ are depicted in Figure S3.

Table S4: Initial guess and optimized parameters for the MM3 discussed in the main text using equations (1+S3+S4+S5) to describe the nonlinear ion-drift-diffusion dynamics. The resulting fitting is depicted on Figure 5(d) in the main manuscript which shows the I-V characteristics of the Au-Ti/TiO_2/Ti-Au device in vacuum and at room temperature. The initial conditions for the dynamical quantities were set at $x(t=0)=0.9$ for the internal state variable, $\tau(t=0)=0.2$ a.u. for the diffusion rate, and $\varepsilon(t=0)=0.05$.

Fitting Parameters	Initial guess	Optimized
α	6.596764	6.84439278
β	0.08313268	0.07331097
γ	0.007	0.00628757
δ	0.635	0.65183438
λ^+	3.965647	4.29791756
λ^-	2.565647	1.92250884
η_1	0.1779536	0.16020241
η_2	0.7847713	0.75022404
η_3	0.2177954	0.25326501
η_4	1.634771	1.49644852
ν	2.26	1.47706601
σ	0.5	0.46579100

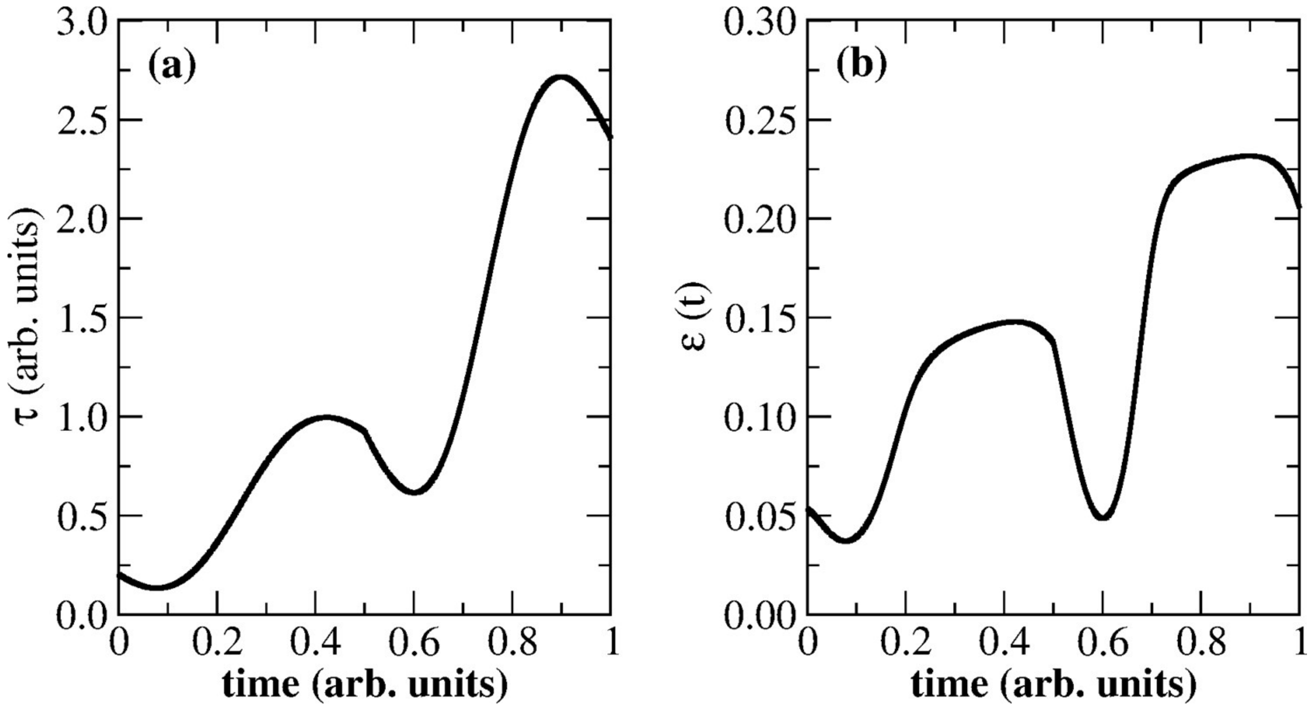

Figure S3: Calculated $\{\tau(t),\varepsilon(t)\}$ (cf. equations (S3) and (S5)) using MM3 with the conditions established on Table S4. This solution was obtained from fitting MM3 onto the I-V characteristics of Au-Ti/TiO_2/Ti-Au devices in vacuum and at room temperature (cf. Figure 5(d) in the main manuscript).

Tables S1, S2, S3, and S4 present the values of the fitting parameters for the four memristive models discussed here and in the main text for the case in which Au-Ti/TiO_2/Ti-Au is in vacuum and at room temperature. We can use some of these parameters to extract useful information about the characteristics of the device interfaces [5, 6], e.g. to estimate the maximum Schottky barrier height, Φ_B [7]. Assuming that the charge transport mechanism of the system at OFF state $(x=0)$ is predominantly due to thermionic emission [8, 9] through the Schottky barrier, the current is then given by

$$I_{TE} = SA^*T^2 e^{\left(-\Phi_B/k_BT\right)} e^{\left(eV/nk_BT\right)}\left[1 - e^{\left(-eV/nk_BT\right)}\right] \qquad (S6)$$

where A^* is the effective Richardson constant, n is the ideality factor, T is the temperature, k_B is the Boltzmann constant, and e is the electron charge. S is the cross-sectional area of the device, in our case, $S = \pi(d/2)^2$ where d is the diameter of the nanowire. For the purpose of estimation, we will consider $A^* \approx A_0 = 1.20173 \times 10^6\ A/m^2K^2$, with A_0 being the universal Richardson constant. By comparing equation (S6) with equation (1) in the main text, for the zero-bias case, we identify

$$\alpha = SA^*T^2 e^{\left(-\Phi_B/k_BT\right)} \qquad (S7)$$

from which the maximum Schottky barrier height can be estimated. Values of α for both memristive descriptions shown in Table S1 result in a Schottky barrier height range of 0.25-0.3 eV using that our nanowire diameters range $d = 50 - 100$ nm. This agrees with existent measured values for other TiO_2-based structures as reported in [10, 11, 12, 13, 14].

The last improvement in the memristive models adopted in this work was done by including a static rectifying current contribution (for thermionic effects) to the total current response function as given in equation (8) in the main text. The initial guess and optimized values for this improved model are shown on Table S5. A sample code for this improved description that reconstructs the I-V fitting of Figure 7 in the main text is provided in the supplemental information [15].

Table S5: Initial guess and optimized parameters for the improved current response function (equation (8) in the main manuscript), with the dynamical variable described by MM1+$^{\tau}$ (equations (1+S1)) to describe the nonlinear ion-drift dynamics. The resulting fitting is depicted on Figure 8 in the main manuscript which shows the I-V characteristics of the Au-Ti/TiO_2/Ti-Au device in vacuum and at room temperature. The initial condition for the internal state variable was set at $x(t = 0) = 0.9$.

Fitting Parameters	Initial guess	Optimized
α_1	8.345194	6.99172421
β_1	0.1078093	0.09768957
α_2	3.345194	5.69422726
β_2	0.11009365	0.01078093
γ	0.005	1.5903×10^{-4}
δ	1.039012	1.00130584
λ^+	3.254168	3.31522859
λ^-	3.254168	3.79686517
η_1	0.26103	0.18971782
η_2	0.3249405	0.38420470
η_3	0.26103	0.18220223
η_4	0.3249405	0.49678829
τ	0.03998672	0.1109234

We tested the robustness of the fitting presented in Figure 7 (main text) by inducing variations in some of the initial values of the parameters in Table S5 and its outcome is shown in Figure S4. The black dots are experimental data and the red surface shell outlines the range where all fitting results - using the last improved memristive model discussed in the main text - fell.

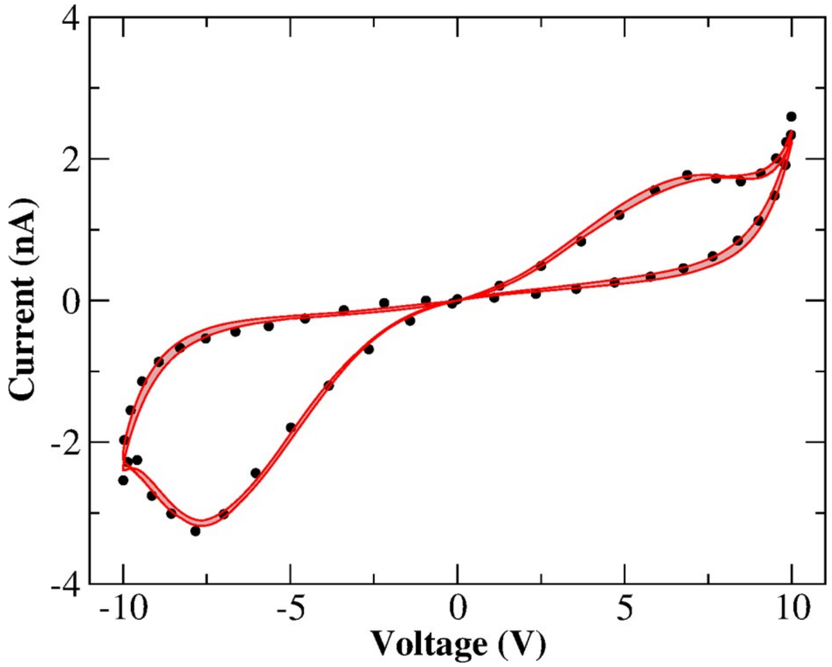

Figure S4: Initial conditions variability tests for the improved memristive model using the response function (8) (main text) with the dynamical variable being described by the nonlinear ion-drift with static diffusion (MM1+τ). The circular symbols are experimental I-V data of our Au-Ti/TiO_2/Ti-Au device in vacuum and at room temperature. (Red surface shell) Variations in the initial values of the parameters shown in Table S5 were induced and all fitting curves fall within the marked region. The fittings follow the dual CW orientation of the I-V hysteresis.

To demonstrate the range of applicability of the model in equation (8) in the main text, we investigated how the shape of the I-V curve of our Au-Ti/TiO_2/Ti-Au device in vacuum can be modified upon independent variation of the parameters presented on Table S5. Figure S5 shows how the I-V hysteresis shape can be modulated by varying a given parameter in the model (while keeping others fixed at their optimum values as on Table S5). The wide variety of I-V shapes displayed in the figure reflects the multiple transport features enclosing this particular Au-Ti/TiO_2/Au-Ti nanowire family that can be captured by our model.

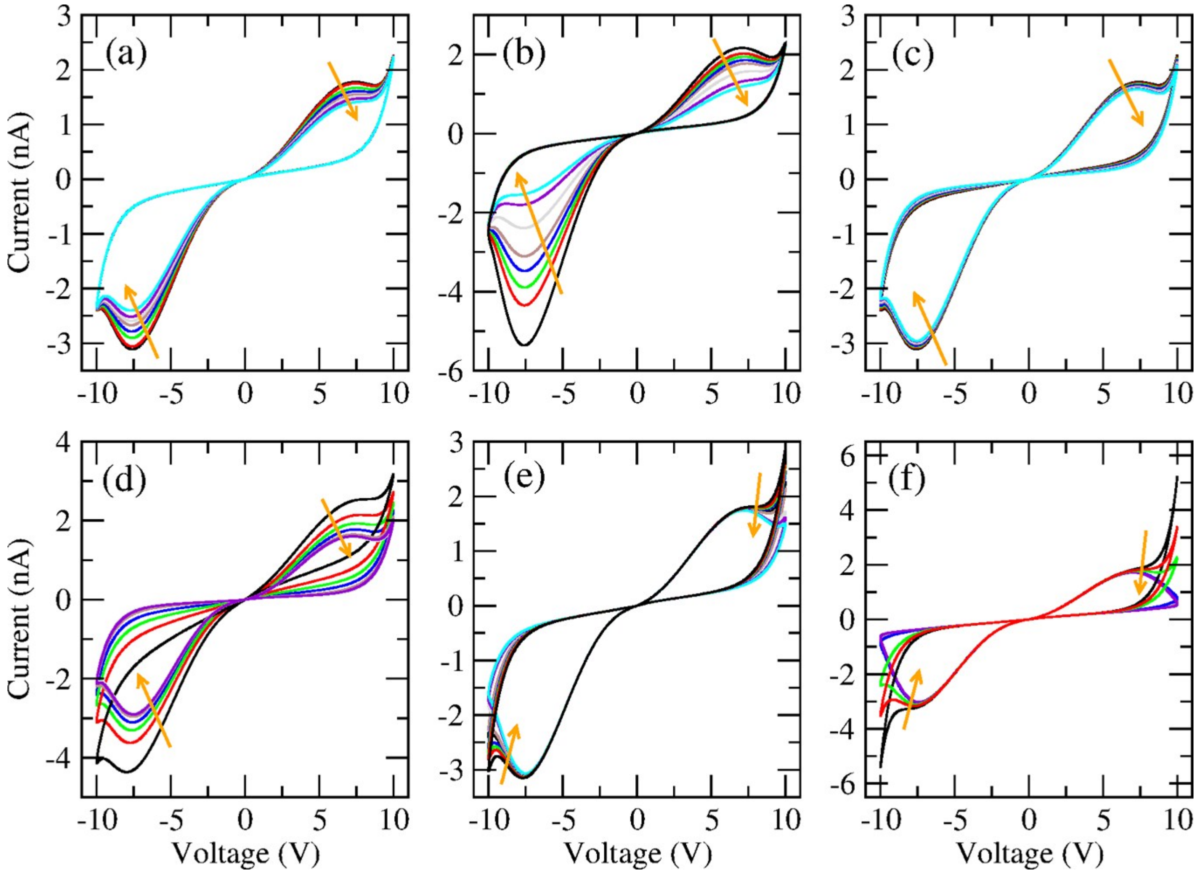

Figure S5: Parameter variability tests for the improved memristive model using the response function (8) (main text) with the dynamical variable being described by the nonlinear ion-drift with static diffusion (MM1+τ). The I-V curves were calculated for the case of the Au-Ti/TiO_2/Ti-Au device in vacuum. All curves follow dual CW orientation. Each panel contains multiple I-V hysteresis loops, each loop corresponds to varying the following parameters individually (while keeping others at their respective optimum values as on Table S5): (a) $\alpha_1 = [5,...,7]$, (b) $\beta_1 = [0.05,...,0.15]$, (c) $\alpha_2 = [2,...,3.5]$, (d) $\beta_2 = [0.005,...,0.015]$, (e) $\gamma = [0.9,...,1.7] \times 10^{-4}$, and (f) $\delta = [0.6,...,1.1]$. The arrows indicate parameters being varied in a decrescent manner.

Finally, Table S6 presents the fitting parameters obtained for the device exposed to air as depicted in Figure 9(b) in the main manuscript. The optimization was done using MM1+$^{\tau}$ description using equation (S1) for [1] nonlinear ion-drift dynamics.

Table S6: Initial guess and optimized parameters for the MM1+$^{\tau}$ discussed in the main text using equations (1+S1) to describe the nonlinear ion-drift dynamics. The resulting fitting is depicted on Figure 9(b) in the main manuscript which shows the I-V characteristics of the Au-Ti/TiO_2/Ti-Au device in air and at room temperature. The initial condition for the internal state variable was set at $x(t=0)=0.9$.

Fitting Parameters	Initial guess	Optimized
α	0.03451944	7.56218566
β	0.1078093	0.00391133
γ	0.01	0.43112914
δ	1.039012	0.45777220
λ	3.254168	19.6579116
η_1	0.26103	0.07417968
η_2	0.3249405	0.05494914
τ	0.03998672	0.02121730

2. Multiple voltage sweeps measurement (testing diffusive effects)

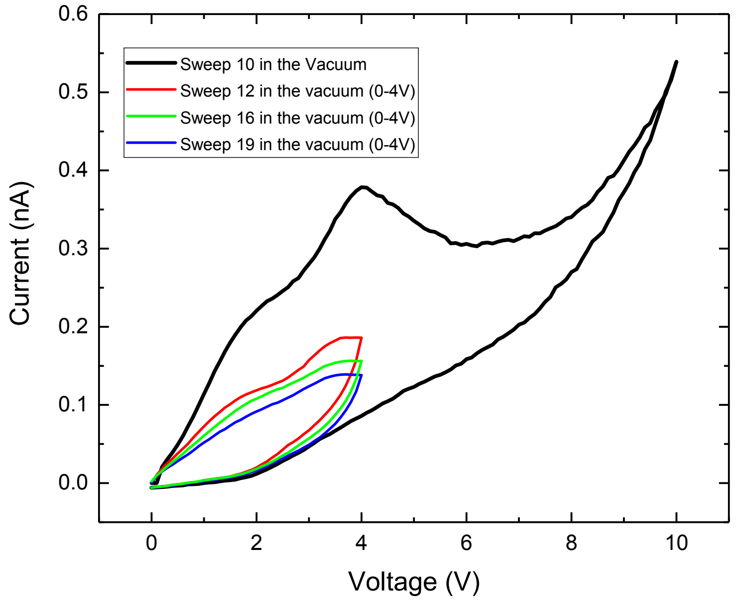

Figure S6: Multiple I-V sweeps of the Au-Ti/TiO_2/Ti-Au structure in vacuum and at room temperature. The direction of all sweeps is CW. The first I-V loop included in the figure is similar to the I-V curve for $V > 0$ presented in Figure 2(d) of the main text with a voltage amplitude sweep of 10 V. Subsequent sweeps were conducted at a smaller bias amplitude of 4 V and caused the dropping drop off the current level and die down of the hysteresis loops.

3. I-V characteristics at distinct temperatures

A detailed investigation on the shape of the I-V characteristics of the Au-Ti/TiO_2/Ti-Au devices in vacuum was carried out in a wide temperature range of 260-370 K with 10 K step differences, which is shown in Figure S7. For all temperatures, a bipolar CW switching with increased hysteresis windows were observed as the temperature increased. The I-V curves evidence temperature dependency in both forward and reverse bias loops. This behaviour can be a result of the diffusivity of oxygen ions (vacancies) which typically depends on the temperature [16]. At low temperatures (< 300 K), the oxygen ions have less mobility, and this would explain the low levels of current found in those I-V curves. At high temperatures, however, the mobility of the oxygen vacancies increases and those can drift rapidly across the bulk of the TiO_{2-x} channel. Current levels increase with temperature with the underlying I-V shape being dictated by the nonlinear ion-drift-diffusion with retention terms.

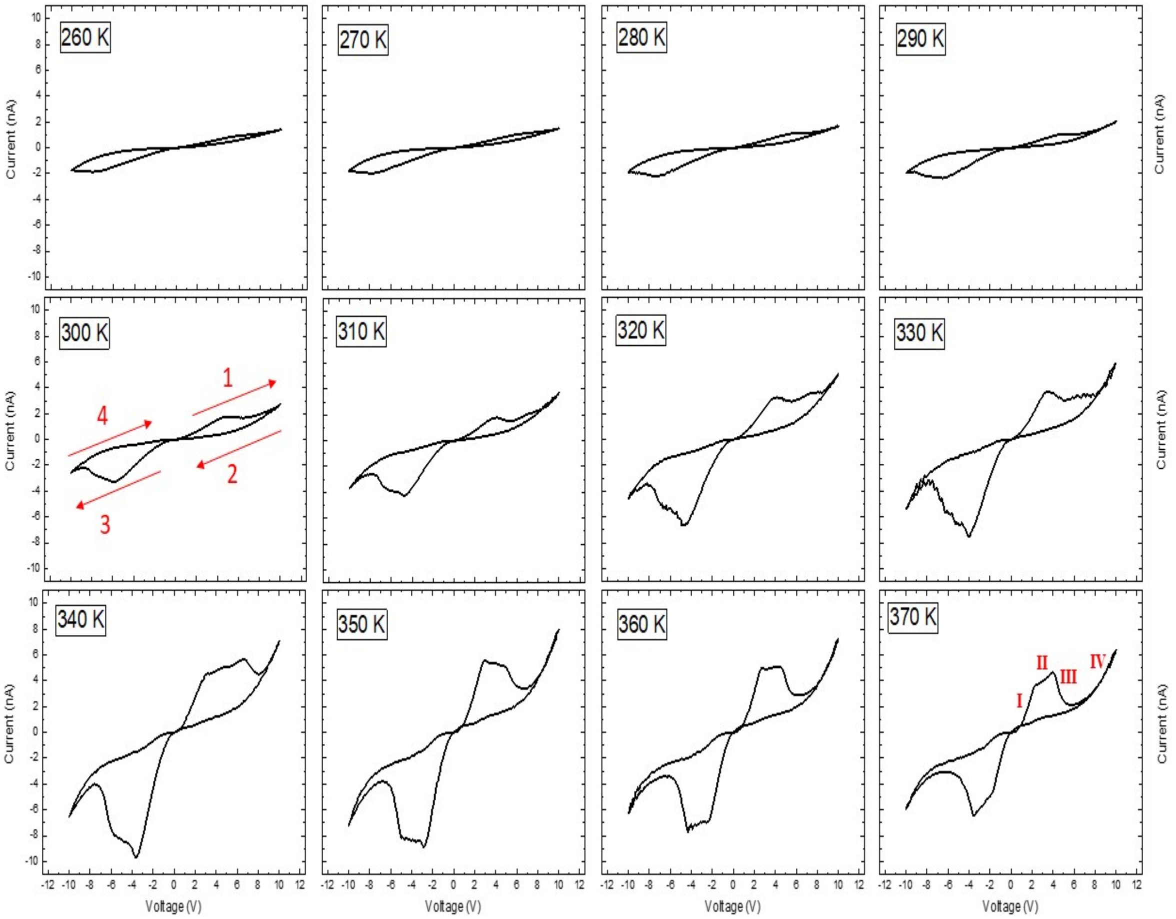

Figure S7: Forward and reverse bias I-V-T characteristics of Au-Ti/TiO_2/Ti-Au device in the temperature range of 260-370 K. The direction of the sweep was the same for all temperatures, CW, and it is highlighted in the 300 K panel with red arrows. The definition of the NDR region enhances with temperature in which well-defined I-V sections can be observed and those are marked with Roman numerals in the 370 K panel.

References:

[1] T. Chang, S.-H. Jo, K.-H. Kim, P. Sheridan, S. Gaba, and W. Lu, "Synaptic behaviors and modeling of a metal oxide memristive device", Appl. Phys. A **102**, 857-863 (2011).

[2] Y. Joglekar, and S. Wolf, "The elusive memristor: properties of basic electrical circuits", European Journal of Physics **30**, 661-675 (2009).

[3] B. Linares-Barranco, and T. Serrano-Gotarredona, "Memristance can explain spike-time-dependent-plasticity in neural synapses." Available from Nature Precedings <http://hdl.handle.net/10101/npre.2009.3010.1> (2009).

[4] E. Zhou, L. Fang, and B. Yang, "A general method to describe forgetting effect of memristors", Phys. Lett. A **383**, 942 (2019).

[5] R. A. Bakar, et al., "Titanium dioxide-based memristive thin film: a correlation study between the experimental work and simulation program with integrated circuit emphasis hyperbolic sine models", Journal of the Electron Devices Society **6**, 1077 (2018).

[6] W. X.-Ping, C. Min, and S. Yi, "Switching mechanism for TiO_2 memristor and quantitative analysis of exponential model parameters", Chin. Phys. B **24**, 088401 (2015).

[7] R. T. Tung, "The physics and chemistry of the Schottky barrier height", Applied Physics Reviews **1**, 011304 (2014).

[8] M. Hansen, M. Ziegler, L. Kolberg, R. Soni, S. Dirkmann, T. Mussenbrock, and H. Kohlstedt, "A double barrier memristive device", Sci. Rep. **5**,13753 (2015).

[9] N. N. K. Reddy, and V. R. Reddy, "Barrier characteristics of Pt/Ru Schottky contacts on n-type GaN based on I–V–T and C–V–T measurements", Bull. Mater. Sci. **35**, 53 (2012).

[10] F. Hossein-Babaei, N. Alaei-Sheini, M. M. Lajvardi, "Oxygen adsorption at noble metal/TiO_2 junctions", IOP Conf. Series: Materials Science and Engineering **108**, 012030 (2016).

[11] L. Michalas, A. Khiat, S. Stathopoulos, and T. Prodromakis, "Electrical characteristics of interfacial barriers at metal-TiO2 contacts", J. Phys. D: Appl. Phys. **51**, 425101 (2018).

[12] S. B. K. Aydin, et al., "ALD TiO_2 thin film as dielectric for Al/p-Si Schottky diode", Bull. Mater. Sci. **37**, 1563 (2014).

[13] M. S. Arshad, et al., "Determination of Schottky barrier height and enhanced photoelectron generation in novel plasmonic immobilized multisegmented (Au/TiO_2) nanorod arrays (NRAs) suitable for solar energy conversion applications", J. Mater. Chem. C **5**, 10509 (2017).

[14] J. Park, K. P. Biju, S. Jung, W. Lee, J. Lee, S. Kim, S. Park, J. Shin, and H. Hwang, "Multibit operation of TiO_x-based ReRAM by Schottky barrier height engineering", IEEE Electron Device Lett. **32**, 476 (2011).

[15] Leader, Jeffery J. (2004), "Numerical Analysis and Scientific Computation", Boston: Addison-Wesley.

[16] J.-K. Lee, et al., "Accurate analysis of conduction and resistive-switching mechanisms in double-layered resistive-switching memory devices", Appl. Phys. Lett. **101**, 103506 (2012).
