# Supplementary material for: Nonlinear ion drift-diffusion memristance description of TiO2 RRAM devices
Source: Nanoscale Adv. 2020 Apr 21;2(6):2514–24. doi: 10.1039/d0na00195c (PMC9419089; doi:10.1039/d0na00195c)

Electronic Supplementary Material (ESI) for Nanoscale Advances.
This journal is © The Royal Society of Chemistry 2020

Supplemental Material: Nonlinear Ion Drift-Diffusion Memristance Description of TiO_2 RRAM Devices (sample code)

```
#!/usr/bin/env python
'''
    File name: testing_params_MM1_tau.py
    Authors: C. G. Rocha, and K. Esteki
    Date created: 10 October 2019
    Date last modified: 06 February 2020
    Python Version: 2.7

    This program is free software: you can redistribute it and/or modify
    it under the terms of the GNU General Public License as published by
    the Free Software Foundation, either version 3 of the License, or
    (at your option) any later version.

    This program is distributed in the hope that it will be useful,
    but WITHOUT ANY WARRANTY; without even the implied warranty of
    MERCHANTABILITY or FITNESS FOR A PARTICULAR PURPOSE. See the
    GNU General Public License for more details.

    You should have received a copy of the GNU General Public License
    along with this program. If not, see <https://www.gnu.org/licenses/>.

    Required packages: numpy, matplotlib, scipy.

    This code is generated for the purpose of testing the output parameters obtained from the
    nonlinear fitting of systems of ODE equations. For this memristive model case (MM1+tau), the
    optimized parameters are lambdax, lambday, eta1, eta2, eta3, eta4, tau, alpha1, beta1,
    gamma, delta, alpha2, beta2, and x0.


Citation:
    If you use this code in academic publications, please, cite our work appropriately.

Parameters:
-----------
   lambdap, lambdan, eta1, eta2, eta3, eta4: float
         Nonlinear ion-drift parameters in the dynamical state equation

   tau: float
         Diffusion rate

   vmax, freq: float
         Voltage amplitude (in Volts) and frequency of the input voltage signal

   alpha1, beta1: float
         Current response function parameters (Schottky contribution)

   gamma, delta: float
         Current response function parameters (tunnelling contribution)

   alpha2, beta2: float
         Current response function parameters (rectifier contribution)

   points: int
         Number of points in the timeline (arb. units)
```

```python

Returns: three plots: (i) x versus t, (ii) I versus V, and (iii) V versus t
'''

# importing canonical python libraries
import matplotlib.pyplot as plt
import numpy as np
from scipy.integrate import odeint

# State equation dx/dt to be integrated
def myFunc(x, t, *args):
    '''After integrated, returns x(t).

    Keyword arguments:
    t -- time
    x -- internal state variable
    *args -- multiple memristive parameters: lambdap, lambdan, eta1, eta2, eta3, eta4, tau
    *args -- input voltage signal parameters: vmax, freq
    '''

    v = vmax * np.sin(2.0 * freq * np.pi * t)
    xdyn = x

    if t <= freq/2:
        dxdt = (lambdap * (np.exp(eta1*v) - np.exp(eta2)) - xdyn/tau) * (1.0 - (2.0*xdyn - 1.0)**2)
    else:
        dxdt = (lambdan * (np.exp(-eta3*v) - np.exp(eta4)) - xdyn/tau) * (1.0 - (2.0*xdyn - 1.0)**2)

    return dxdt


# general parameters for the voltage (sinusoidal) input
vmax = 10.0
freq = 1.0

# parameters taken from the fitting procedure
alpha1 = 6.82991497
beta1 = 0.10051066
alpha2 = 3.42451238
beta2 = 0.01325593
gamma = 1.6138e-04
delta = 0.99905798

lambdap = 3.05688105
lambdan = 3.56661637
eta1 = 0.19781416
eta2 = 0.35545468
eta3 = 0.19863980
eta4 = 0.53302178
tau = 0.10820846
dxdt_params = (vmax, freq, lambdap, lambdan, eta1, eta2, eta3, eta4, tau)

# number of points in the timeline
points = 10000

# create a timeline vector
t = np.array([0.0, 1.0])
tspan_vector = np.linspace( t[0], t[1], points)

```

```
# voltage input signal
V = vmax * np.sin(freq * 2.0 * np.pi * tspan_vector)

# steps in the timeline
h =  tspan_vector[1] - tspan_vector[0]

# initial conditions for the internal state variable
x0 = 0.9

# integration of the dynamical state equation
xs = odeint(myFunc, x0, tspan_vector, args=dxdt_params)

# calculating current values from the solution x(t)
ic = (1.0 - xs[:,0]) * alpha1 * (1.0 - np.exp(-beta1 * V[:])) + xs[:,0] * gamma * np.sinh(delta * V[:]) +
    alpha2 * (1.0 - np.exp(-beta2 * V[:]))

# plotting instructions

# plotting x versus t
plt.figure()
plt.plot(tspan_vector, xs[:,0], 'r')
plt.xlim(t[0], t[1])
plt.xlabel('t', fontsize=17)
plt.ylabel('x', fontsize=17)
plt.tight_layout()

# plotting I versus V
plt.figure()
plt.plot(V, ic, 'r')
plt.xlabel('V', fontsize=17)
plt.ylabel('I', fontsize=17)

# plotting V versus t
plt.figure()
plt.plot(tspan_vector, V, 'r')
plt.xlabel('t', fontsize=17)
plt.ylabel('V', fontsize=17)

plt.show()
```

The figures below are screenshots of the output of this sample code for the sake of reference. The code plots $x(t) \times t$, $I \times V$, and $V(t) \times t$. The used parameters were optimized to fit our particular experimental data of Au-Ti/TiO_2/Ti-Au devices in vacuum and at room temperature. These parameters work better for a single voltage cycle.

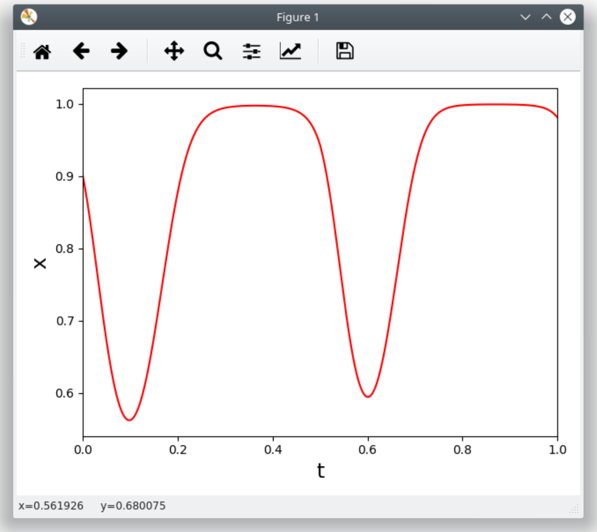

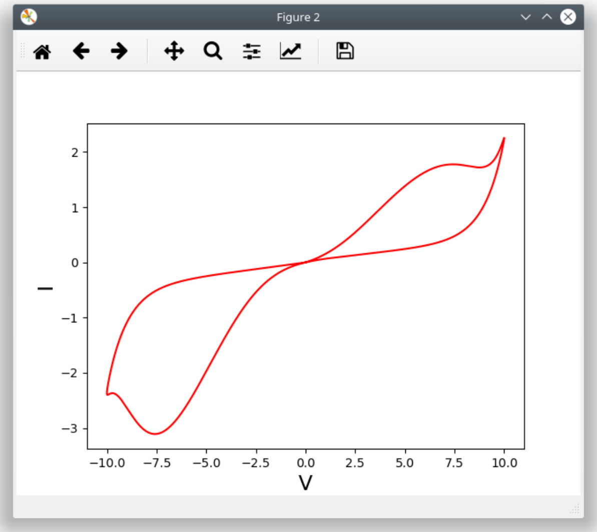

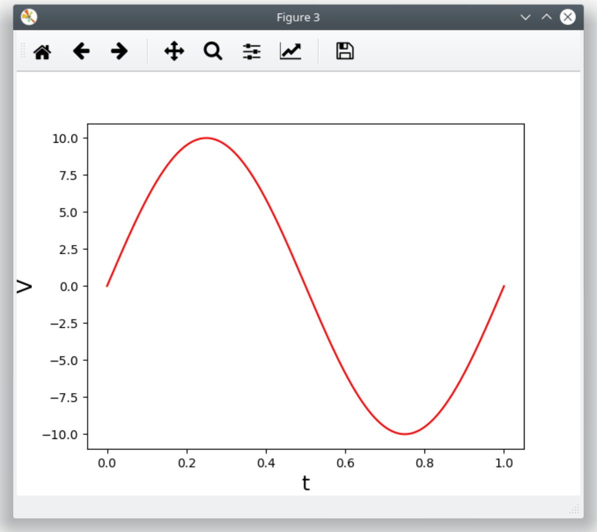

Supplement: NA-002-D0NA00195C-s002 [file NA-002-D0NA00195C-s002.pdf]
